# Nationwide analysis of air pollution hotspots across India: A spatiotemporal PM_2.5_ trend analysis (2008–2019)

**DOI:** 10.1016/j.envres.2024.120276

**Published:** 2024-11-05

**Authors:** Suganthi Jaganathan, Ajit Rajiva, Heresh Amini, Jeroen de Bont, Shweta Dixit, Anubrati Dutta, Itai Kloog, Kevin J. Lane, Jyothi S. Menon, Amruta Nori-Sarma, Dorairaj Prabhakaran, Joel Schwartz, Praggya Sharma, Massimo Stafoggia, Gagandeep Kaur Walia, Gregory A. Wellenius, Poornima Prabhakaran, Petter Ljungman, Siddhartha Mandal

**Affiliations:** a Institute of Environmental Medicine, Karolinska Institutet, Stockholm, Sweden; b Centre for Health Analytics Research and Trends, Ashoka University, Sonipat, Haryana, India; c Centre for Chronic Disease Control, Delhi, NCR, India; d Department of Geography and Environment, Faculty of Humanities and Social Sciences, Ben-Gurion University of the Negev, Beer-Sheva, Israel; e Department of Environmental Medicine and Public Health, Icahn School of Medicine at Mount Sinai, New York, NY, USA; f Department of Environmental Health, School of Public Health, Boston University, Boston, MA, USA; g Department of Environmental Health, Harvard T.H. Chan School of Public Health, Boston, MA, USA; h Department of Epidemiology, Lazio Region Health Service / ASL Roma 1, Rome, Italy; i Department of Cardiology and Clinical Physiology, Danderyd University Hospital, 182 57, Danderyd, Sweden

**Keywords:** Air pollution, Hotspot analysis, Fine particulate matter, Spatiotemporal analysis, India, PM2.5

## Abstract

**Introduction:**

India experiences high levels of air pollution as measured by fine particulate matter *<*2.5 μm (PM_2.5_) across the country. With limited resources, it is imperative to identify the most impacted areas. We aimed to identify air pollution hotspots in India and analyze temporal trends.

**Methods:**

We conducted a geospatial analysis using Getis-Ord Gi statistics on gridded-annual levels of PM_2.5_ disaggregated for every state/UT and three largest cities [Delhi, Kolkata & Mumbai] of India from 2008 to 2019. The annual average PM_2.5_ was derived from a validated and robust nationwide spatiotemporal model (1km×1km). Hotspots were identified annually using $\text{G}{\text{i}}^{*}$ score and p-value and temporal trends across 2 periods [T1:2008–2013 & T2:2014–2019] for each spatial unit. We classified temporal trends based on the number of occurrences of hotspots in T1 and in T2 as consistent (similar in T1 & T2), declining (decreasing in T2) and emerging (increasing in T2) hotspots.

**Results:**

We identified consistent hotspots in 9.9% followed by emerging hotspots in 2.6% of the country where 16% and 4.9% people live. In addition, we identified declining hotspots in 2.6% area with 3.4% of the population. Rajasthan had largest share of area identified as consistent hotspots while Uttar Pradesh had densely populated consistent hotspots. Maharashtra had both higher number of areas identified as emerging and declining hotspots. Among the largest cities, Kolkata had highest proportion of consistent hotspots. We identified 170 additional cities with either consistent or emerging hotspots beyond the non-attainment cities as defined by the National Clean Air Programme.

**Conclusion:**

India continues to have large areas of consistent and emerging hotspots of air pollution where close to a fifth of India’s population live. Identifying hotspots can inform strategic approach for targeted action in air quality management, appropriate resource allocation and a baseline for assessing intervention effectiveness and future programs and policies, including health.

## 1. Introduction

A wealth of evidence supports that ambient air pollution, especially fine particulate matter *<*2.5 μm (PM_2.5_), is a global problem contributing to premature mortality and diseases (U.S. EPA, 2019). In India, home to 1.4 billion people (State of World Population, 2024–Interwoven Lives, 2024), the PM_2.5_ concentrations routinely exceed both national ambient air quality guidelines (NAAQS) and World Health Organization (WHO) guideline levels by wide margins. The Global Burden of Disease Study (GBD) 2019 estimated that ~1 million deaths in India were attributable to ambient air pollution in 2019 (Pandey et al., 2021). In support of this, there is a growing evidence base of studies from India demonstrating associations between short-term and long-term exposure and disease burden and mortality (Pandey et al., 2021; Prabhakaran et al., 2020; Mandal et al., 2023; Krishna et al., 2021; Anand et al., 2024; de Bont et al., 2024; Brown et al., 2022; Nair et al., 2021; George et al., 2024).

However, the current estimates of the disease burden in India provide limited actionable insights for policymakers, given the coarse spatial resolution and yearly time scale of existing PM_2.5_ estimates. Spatial variation in PM_2.5_ levels are an important driver of differences in air pollution-related health effects and mortality both globally and in India (Liu et al., 2021; Khomenko et al., 2023; Pinto et al., 2004; Tiwari et al., 2023; Jia et al., 2021). Given that spatial differences are likely to be caused by local sources, this represents an important and potential area for effective public policy and intervention programs. The widely used GBD models based on exposure assessment done at 11kmx11km do not have the granularity to identify PM_2.5_ levels at a finer resolution impeding the identification of local sources.

Previous efforts have used hotspot analyses to identify environmentally sensitive, industrial, or densely populated areas for common pollutants such as NO_2_ and PM_2.5_ in China, Portugal, and the US (Ruidas and Pal, 2022; Montgomery et al., 2023; Ali et al., 2023; Abecasis et al., 2022). In India, initial work to identify hotspots were limited to exploring intra-urban air pollution hotspots in and around Delhi based on daily data from the monitoring stations (Goyal et al., 2021). Another study conducted in Delhi, based on only a two-year period, used machine learning techniques to identify local PM_2.5_ hotspots/cool spots at a 300m spatial resolution using satellite imagery, monitor data, and meteorological information (Zheng et al., 2021). One study covering three years highlighted the influence of regional sources of crop burning on seasonal increases in PM_2.5_ for the Delhi (Kumar et al., 2024). Finally, a study from 2012 using a prediction model with very coarse spatial resolution analyzed hotspots on a national level. This study identified regional hotspots using the air pollution data but could not pinpoint hotspots on a finer spatial scale (Dey et al., 2012).

Visualizations showing how PM_2.5_ varies locally within a city and identifying temporal patterns would inform new policies to prioritize the most impacted areas. To overcome the spatial and temporal limitations of the air pollution monitoring network and some of the limitations of previous prediction models, we developed a machine learning-based ensemble model estimating daily levels of PM_2.5_ across India over 13 years (2008–2020) at a fine spatial resolution of 1kmx1km using a comprehensive set of spatial and temporal predictors (Mandal et al., 2024). In contrast to other attempts to predict PM_2.5_ in India beyond the monitoring network, our method developed by Mandal et al. incorporated a multitude of spatiotemporal predictors of PM_2.5,_ such as land use patterns and meteorology, with less reliance on aerosol optical depth and missing rural and peri-urban representation. Further, unlike existing models, the model was calibrated against a much larger number of ground-monitoring observations using a robust validation technique.

In this study, we aimed to assess 1) Spatial distribution of hotspots at both a) the state-/union territory- and b) city-levels. 2) To analyze their temporal trends to identify consistent, emerging and declining hotspots across the country. 3) Describe the population exposure at different types of hotspots and common sources of air pollution.

## 2. Methods

### 2.1. Study area: states/union territories and major cities in India

All 34 states/union territories (UT) except Andaman, Nicobar and Lakshadweep Islands were studied individually at 1kmx1km spatial units from the period 2008 to 2019 (12 years), covering both rural and urban areas. Three largest cities, including Delhi, Kolkata, and Mumbai, were also studied during the same period. We used population density (1 sq. km) from the Socioeconomic data and Application Center website (Center for International Earth Science Information Network - CIESIN - Columbia University, 2018) for 2015 to describe the population at the identified hotspots. We also used point sources data for location of major cities (Location of major Indian cities, 2019), toll plazas (Toll plazas), powerplants and the primary fuel type (Powerplants, 2024), airports (Airports: David Megginson, 2008) and road network (Road network) to add contextual information for the study areas.

### 2.2. Exposure assessment

We obtained annual average PM_2.5_ concentrations at 1 km × 1 km spatial resolution across India using an ensemble averaging approach for the study period. A detailed explanation of the modelling approach is described elsewhere (Mandal et al., 2024). Briefly, we collected ground monitoring-based observations of daily average PM_2.5_ and PM_10_ across 1056 locations and an extensive set of predictors encompassing satellite-based observations, meteorology, land-use patterns, emissions inventories, and reanalysis-based data. Further, unlike existing models, the model was calibrated against a much larger number of ground-monitoring observations using a robust validation technique that left out 20% monitors while training the model, stratified across quintiles of data contribution and zone of the country ensuring representatives of training and test datasets. We trained four machine learning methods (deep learning, random forests, gradient boosting, and extreme gradient boosting) on the remaining monitors. The optimized models were implemented on the left-out validation data to obtain learner-specific predictions and combined using a Gaussian process regression to obtain the final predictions. This methodology allowed us to obtain PM_2.5_ exposures in areas where there was no monitoring data available. Cross-validated prediction accuracy for annual averages was very high (R^2^_=_ 0.94, mean absolute error (MAE) = 8.8 μg/m^3^).

### 2.3. Spatial analysis

A hotspot can be defined as an area that has significantly higher count/concentration of pollution levels compared to the expected number given a random distribution of count/concentration when compared to its spatial neighbors (Getis and Ord, 1992). There are several existing methods for analyzing spatial patterns and detecting hotspots using global spatial autocorrelation statistics such as global Moran’s I and global Geary’s I and local indicators of spatial association (LISA) such as Getis-Ord $\text{G}{\text{i}}^{*}$ statistic. Global spatial autocorrelation statistics measure spatial association across the entire region under study. When compared to the global spatial autocorrelation, LISA has been widely used for identifying spatial clusters and outliers at fine spatial scale. Hence $\text{G}{\text{i}}^{*}$ statistic is also known as hotspot analysis, an appropriate method/statistical analysis tool to address the knowledge gap. The method evaluates the degree to which each feature is surrounded by features with similarly high or low values within a specified geographical distance (neighborhood). Below, we describe the steps taken to perform this analysis.

#### Step 1. Creating neighbor sets

In total, for the whole country, we had n = 3,675,744 grids. We then removed the grids (n = 5848, 0.15% of total) with missing data on predictors or coastal areas or some areas over the water bodies. Following this, we created spatial neighbors (grids with adjoining boundaries) for each studied grid, checked for empty neighbor sets, and removed polygons with empty neighbor sets from the data. We repeated this step for each state/UT and city that was studied.

#### Step 2. Assigning spatial weights to neighbors

Next, we identified neighbors with queen contiguity, i.e., all the grid cells sharing borders with the grid that is examined. Then, we assigned binary weighting, with a weight of “1” for neighboring grids (grids that share borders) and a weight of “0” for all other grids that don’t share borders.

This spatial weighting is based on Tobler’s first law of geography, (Tobler’s law) (Tobler, 1970) that states “everything is related to everything else, but near things are more related than distant things” and is the fundamental assumption used in all spatial analysis.

#### Step 3. Spatial analysis using $\text{G}{\text{i}}^{*}$ test

As a final step of spatial analysis, we conducted the local $\text{G}{\text{i}}^{*}$ test with simulations (n = 99) and classified the grids based on the $\text{G}{\text{i}}^{*}$ score and p-value for each year.

To compute the $\text{G}{\text{i}}^{*}$ score for a given grid cell ‘i’:

$$Gi^{*}=\frac{\sum_{j=1}^{n}w_{i,jxj}-\bar{X}\sum_{j=1}^{n}w_{i,j}}{S\sqrt{\frac{\left[n\sum_{j=1}^{n}w_{i,j}^{2}-{\left(\sum_{j=1}^{n}w_{i,j}\right)}^{2}\right]}{n-1}}}$$

$$\bar{X}=\frac{\sum_{j=1}^{n}x_{j}}{n}$$

$$S=\sqrt{\frac{\sum_{j=1}^{n}{x_{j_{j}}}^{2}}{n}}-{\left(\bar{X}\right)}^{2}$$

where x_j_ is the PM_2.5_ concentration for grid cell j and n is the total number of grid cells within a particular spatial unit. We calculate the spatial weight between grid cells i and j using the Queen’s Contiguity method (w_ij_ = 1, if two grid cells are adjacent and 0 otherwise).

We performed Monte Carlo randomization (n = 99) to estimate the sampling distribution and check the robustness of the $\text{G}{\text{i}}^{*}$ scores. The $\text{G}{\text{i}}^{*}$ statistic computes a Z-score for each grid cell and identifies areas where a grid cell and its neighbors’ values are significantly higher or lower than would be expected if values were distributed randomly across space.

#### Step 4. Classification of hot and cold spots

This process was repeated individually for each spatial unit (state/UT/city) and every year to classify as a hotspot (statistically significant higher $\text{G}{\text{i}}^{*}$ score with significance testing of p *<* 0.05 at the 95% confidence level), coldspot (statistically significant lower $\text{G}{\text{i}}^{*}$ score with significance testing of p *<* 0.05 at the 95% confidence level), or inconsistent (no significant difference in levels compared to neighbors and the confidence level below p *<* 0.05) (Getis and Ord, 1992; Ord and Getis, 1995). The $\text{G}{\text{i}}^{*}$ score is sensitive to the spatial extent being considered, indicating hotspots relative to the distributions within the spatial extent assessed. We performed these analyses at each state/UT/city to identify hyperlocal hotspots and provide actionable information to policymakers at these levels of government. On a national scale, hotspot analyses provide little additional information compared to absolute levels and are unable to highlight hyperlocal accumulations.

#### Step 5. Identifying temporal trends of the distribution of hot and cold spots

**Fig. 1.**
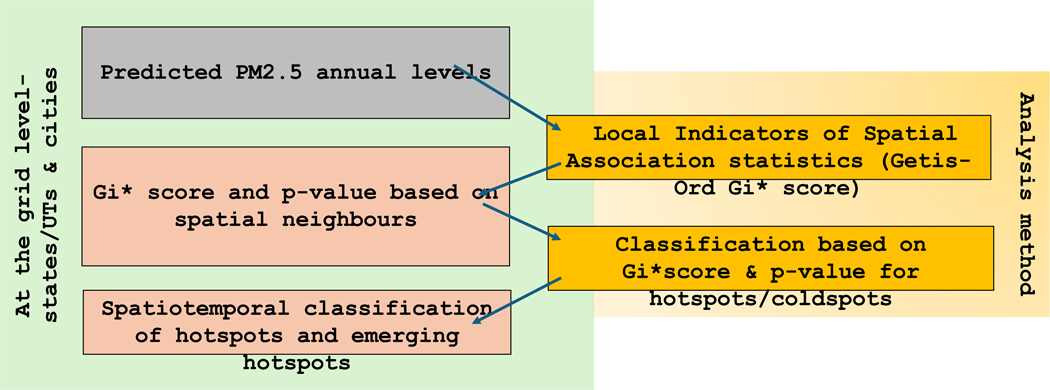
Method for hotspot identification among states/UTs and cities.

After assigning hotspots across the grid cells for each year, we divided our dataset into two periods to identify the temporal clustering of hotspots over the years 2008–2013 (T1) and 2014–2019 (T2). Since we had 12 years of PM_2.5_ data, we divided our dataset into two equal periods to observe any specific temporal clustering of these hotspots. For each time period (T1 & T2), we counted the times a grid was assigned as a hotspot and classified the grids based on the count between T1 and T2: Consistent hotspots were defined as hotspots for ≥5 years in 1 and ≥ 3 years in T2, capturing grid cells that were almost always significant in period 1 and at least half of the time also significant in the second period. Declining hotspots were defined as hotspots for ≥3 years in T1 & ≤2 years in T2, meaning grid cells that at least half of the time identified as significant hotspots during the first period but only at most one third of the time identified as a hotspot during the second half, indicating improving levels. Emerging hotspots were defined as hotspots for ≤3 years in T1 and ≥4 years in T2, capturing grid cells that were only identified as significant hotspots up to half of the years in the first period but at least two thirds of the years in the second period. All others that didn’t meet the criteria set by the authors were termed inconsistent for this study. (Fig. 1). The goal of hotspot analysis is to identify local areas that are more polluted than their neighbors, rather than regional areas and their development across the two time periods of study. Local hotspots, compared to neighboring areas, may result from local pollutant sources.

We are reporting the results of the state/UT-level analyses from the country overall and in specific details for three zones selected to represent more than 60% of the country’s population. This included the North zone: States: Rajasthan, Haryana, Punjab & Himachal Pradesh; Union territories: Delhi, Chandigarh & Jammu and Kashmir; the Central zone: Uttarakhand, Uttar Pradesh, Madhya Pradesh & Chhattisgarh; and South zone: Sates: Tamil Nadu, Telangana, Andhra Pradesh, Kerala & Karnataka; Union territory: Puducherry. Zonal maps are provided in the supplement for the zones not included in the manuscript. Furthermore, we present city-level analyses from the three largest cities: Delhi, Kolkata Metropolitan region and Mumbai. All spatiotemporal analyses were conducted using R statistical software version 4.1.2, and the following packages were used: ’sf’, ’spdep’,’rgdal’, ’tidyverse’, ‘sfdep’ and ’ggplot2’ for the figures. The maps were created in QGIS version 3.28. This work was approved by the Health Ministry Screening Committee at the Indian Council of Medical Research and the ethics committee at Ashoka University.

## 3. Results

### 3.1. Absolute PM_2.5_ levels across the country

**Fig. 2.**
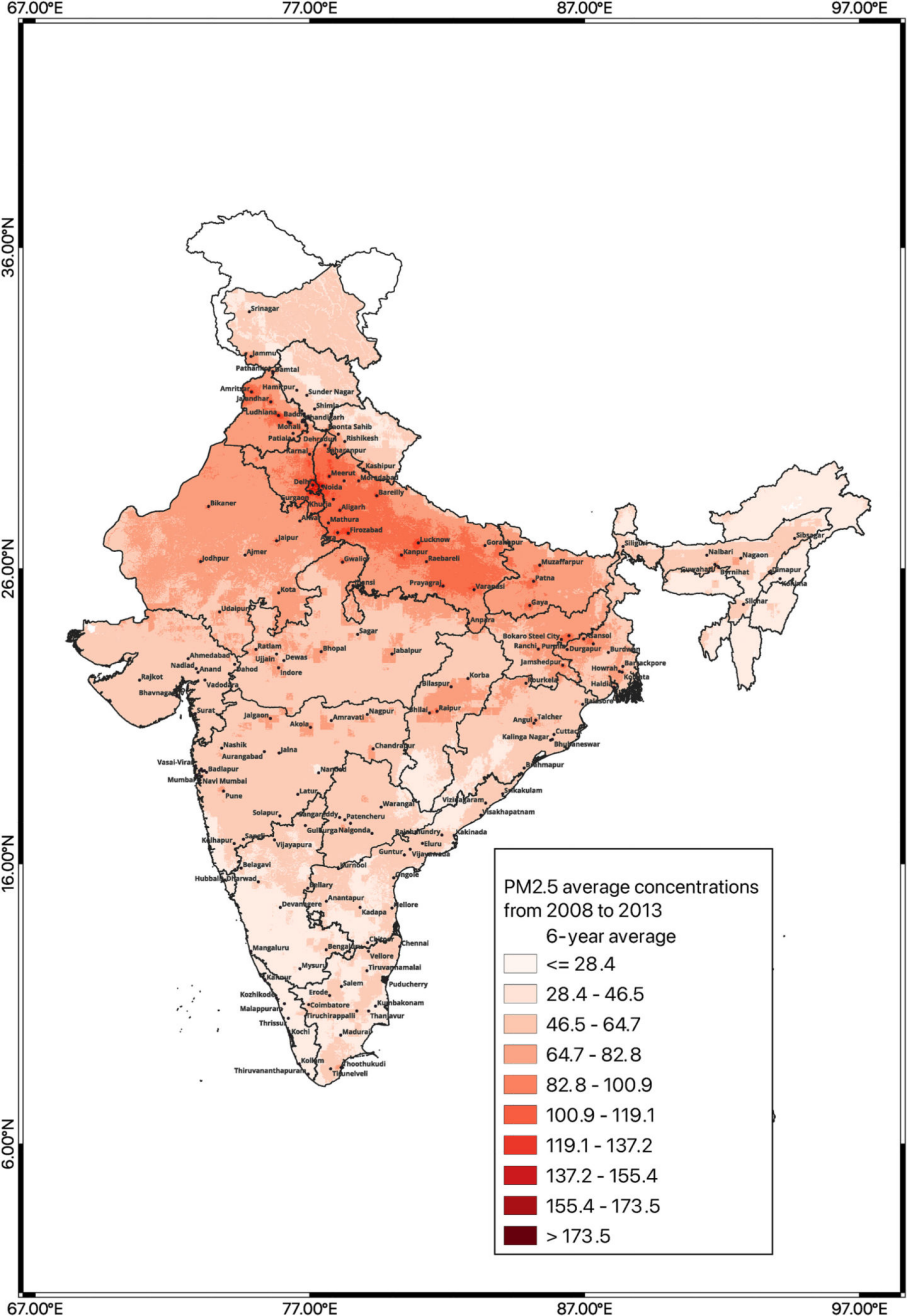

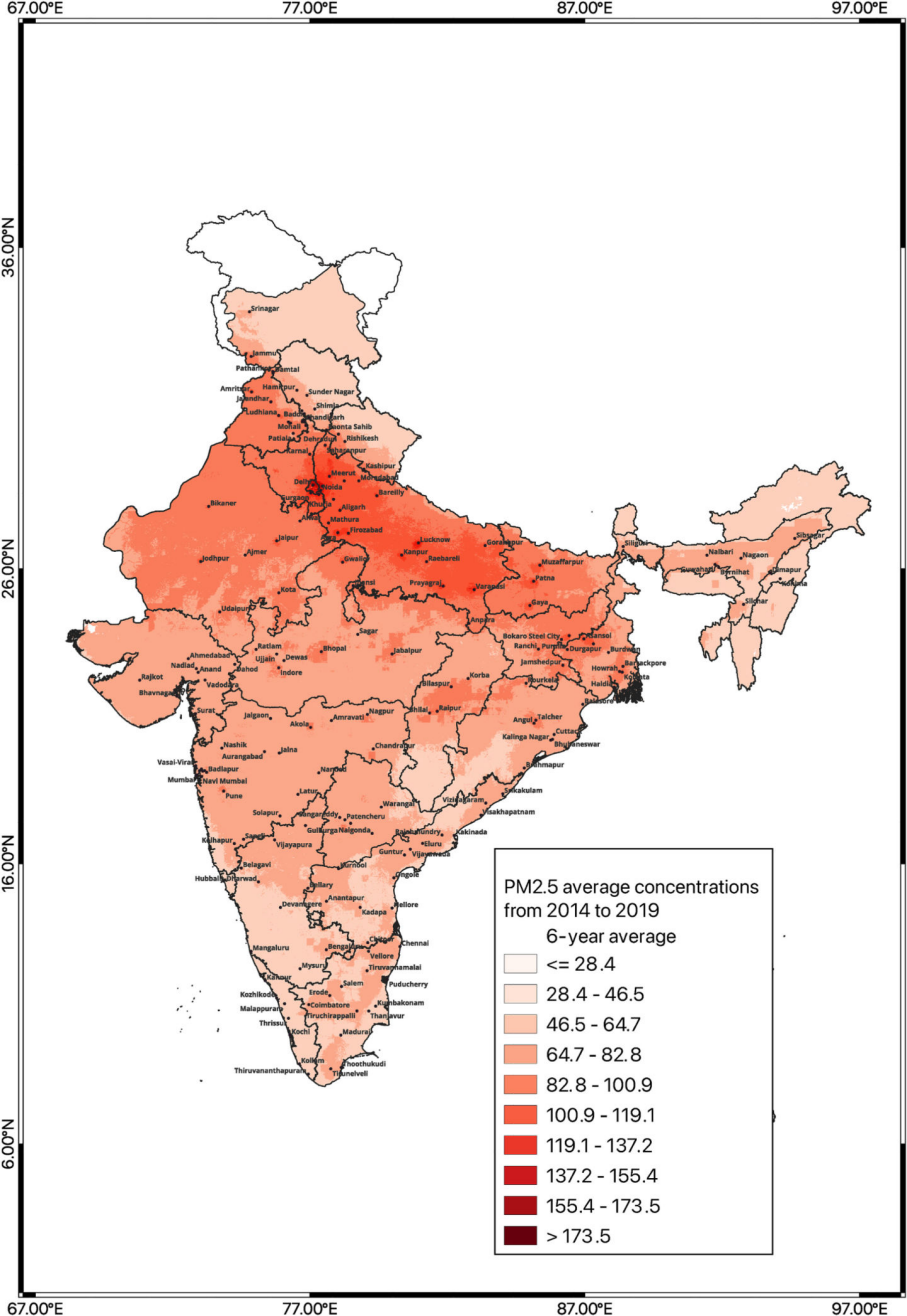

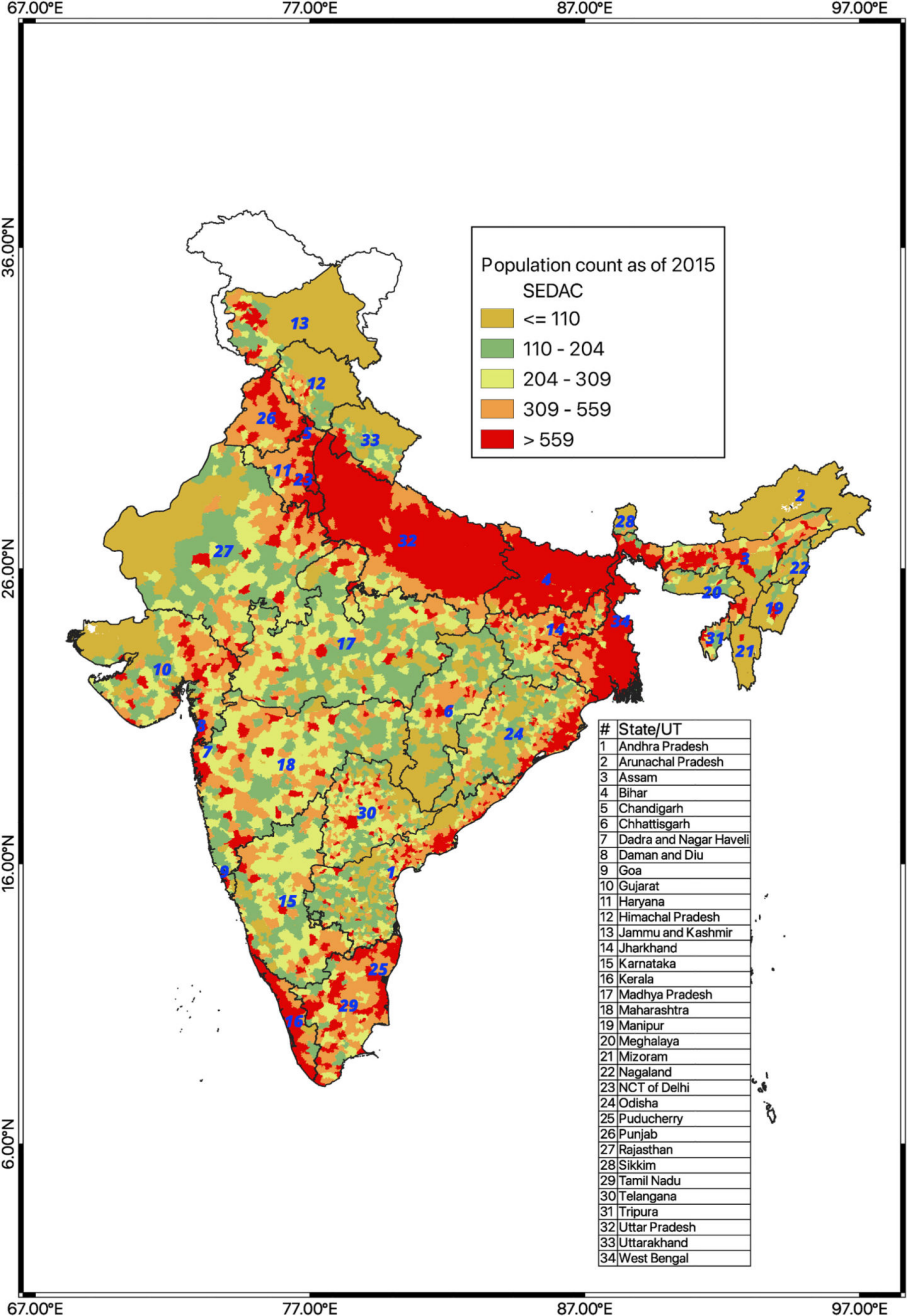
Six-year averages of PM_2.5_ concentrations across India using daily average PM_2.5_ predictions from an ensemble averaging based spatiotemporal model (Mandal et al., 2024). Fig. 2a 6-year average from 2008 to 2013; Fig. 2b 6-year average from 2014 to 2019 & Fig. 2c Gridded Population from SEDAC for 2015 & italicized number in blue colour on each state/UT indicates each state’s location according to the key provided on the right side.

PM_2.5_ levels in India showed a clear increase between the first half of the study period and the second half as well as a robust spatial pattern with the highest averages in the northern area of the densely populated Indo-Gangetic plain stretching from northwest to southeast along the southern border of the Himalayan Mountain range with particularly high levels in and around Delhi (Fig. 2). This corresponds to the states in the Central and Northern zones, whereas the lowest PM_2.5_ averages were observed across Southern, Western, and North-eastern states. State-wide 12-year averages of PM_2.5_ concentrations (standard deviation [SD]) (all states excluding Andaman, Nicobar and Lakshadweep Islands), ranged from 17.8 μg/m^3^ [0.9] in Mizoram to 83.0 μg/m^3^ [9.7] in Uttar Pradesh (Supplementary Table S1). The state of Haryana had the highest standard deviation (SD) of 10.6 μg/m^3^ among all states studied, followed by Uttar Pradesh with 9.7 μg/m^3^ and Punjab with 8.2 μg/m^3^ indicating high within-state spatial variability. Delhi, a union territory and country’s capital city, had the highest levels of PM_2.5_ for a 12-year average at 107.1 μg/m^3^ [SD = 13.6].

### 3.2. Identified hotspots across the country

**Fig. 3.**
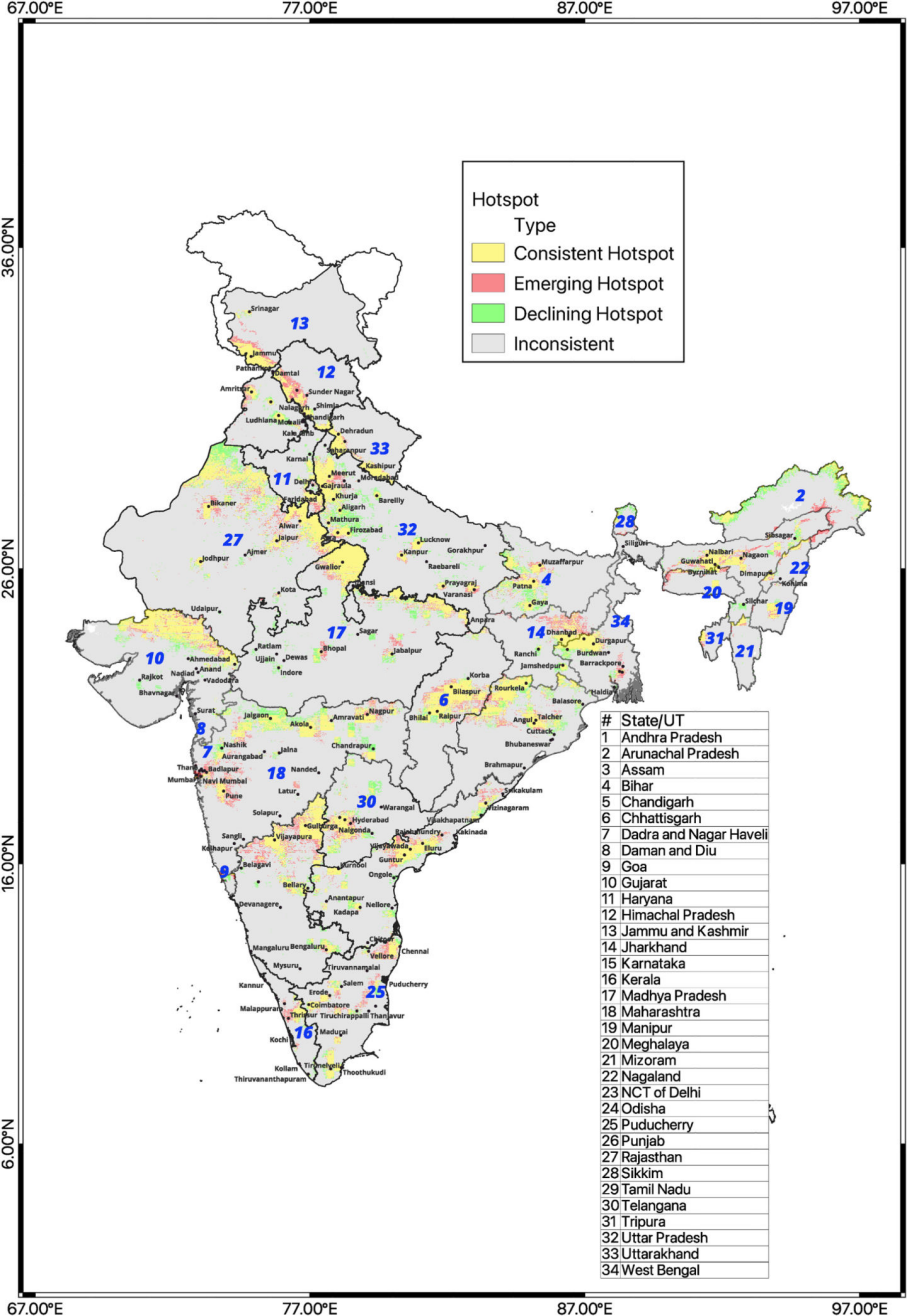
National hotspots trends: Adjoined state/UT-level analysis across 12 years [2008–2019] using Getis-Ord $\text{G}{\text{i}}^{*}$ statistic at a = 0.05 significance level. The italicized number in blue colour on each state/UT indicates each state’s (UT) location according to the key provided on the right side.

State-level hotspot analyses (including union territories) identified spatially clustered grids within each state/UT with consistently high, emerging high and declining PM_2.5_ levels between the two time periods of analysis, both in and outside urban centers (Fig. 3). Notably, these include locations outside of major cities, with less monitoring. The analyses provided evidence of local patterns of exposure suggesting local source emissions.

**Fig. 4.**
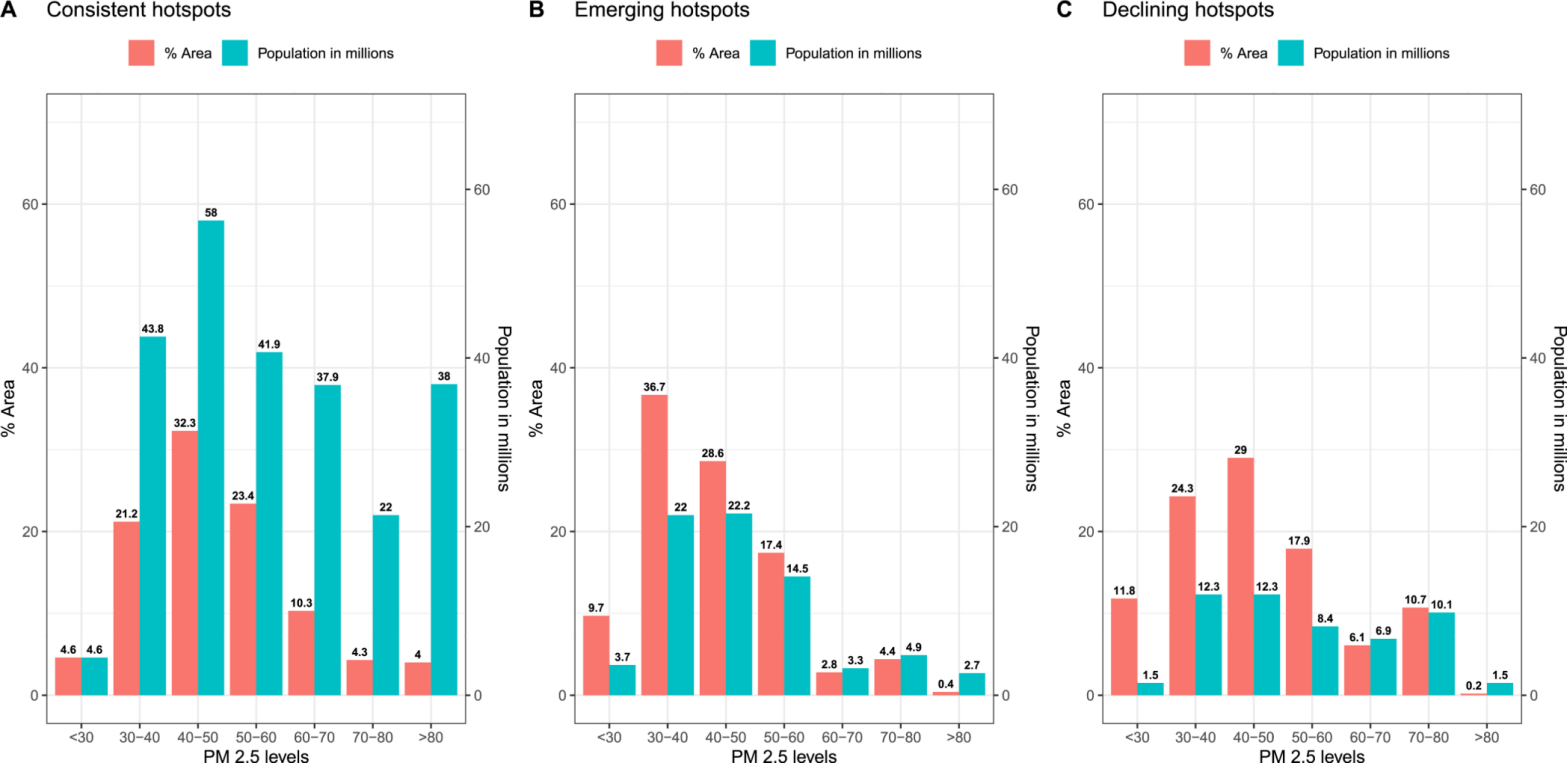
a, b & c: Bar chart on consistent, emerging, and declining hotspots from the state-level analyses. Y-axis indicates %area [left] and population in millions [right] across X-axis of 12-year average PM_2.5_ distribution].

Summarizing state-level and union territories’ analyses, we identified consistent hotspots covering 9.9% of the country’s area including a total of about 250 million inhabitants (16.2% of the country’s population). Additionally, 2.6% of the country’s area was identified as emerging hotspots including 75.3 million inhabitants [4.9%] and a similar area of 2.6 % as declining hotspots but with fewer (52 million, 3.4%) inhabitants [Supplementary Table S1]. In our state-level hotspots analyses, the highest number of grids identified as consistent and declining hotspots (i.e. local/hyper-local areas significantly more polluted than the state/UT as a whole) were found in the central zone (Uttarakhand, Uttar Pradesh, Madhya Pradesh & Chhattisgarh) followed by the southern zone (Tamil Nadu, Telangana, Andhra Pradesh, Puducherry, Kerala & Karnataka). Emerging hotspots were highest in number in the southern zone followed by the northern zone (Rajasthan, Haryana, Punjab, Delhi, Himachal Pradesh & Jammu and Kashmir). The largest number of people living in consistent and declining hotspots was found in the central zone. Among the emerging hotspots, the highest numbers lived in the eastern zone followed by the southern zone. Over 74% of the consistent hotspots had 12-year averages *>*40 μg/m^3^ exceeding the Indian national ambient air quality standards (NAAQS) including around 197 million inhabitants (Fig. 4a). More than 50% of the consistent hotspots had PM_2.5_ averages between 30 and 50μg/m3 and included 100 million inhabitants. Around 18% of the consistent hotspots demonstrated PM_2.5_ averages over 60μg/m3 and included 97.9 million inhabitants. Compared to the consistent hotspots, emerging hotspots showed a roughly similar range across PM_2.5_ concentrations and fewer exposed population although, as expected slightly lower averages (Fig. 4b). Declining hotspots included around 45 million persons exposed and the majority of declining hotspots had 12-year average concentrations around 30–60μg/m3 (Fig. 4c).

### 3.3. Hotspot trends from select zones of India

**Fig. 5.**
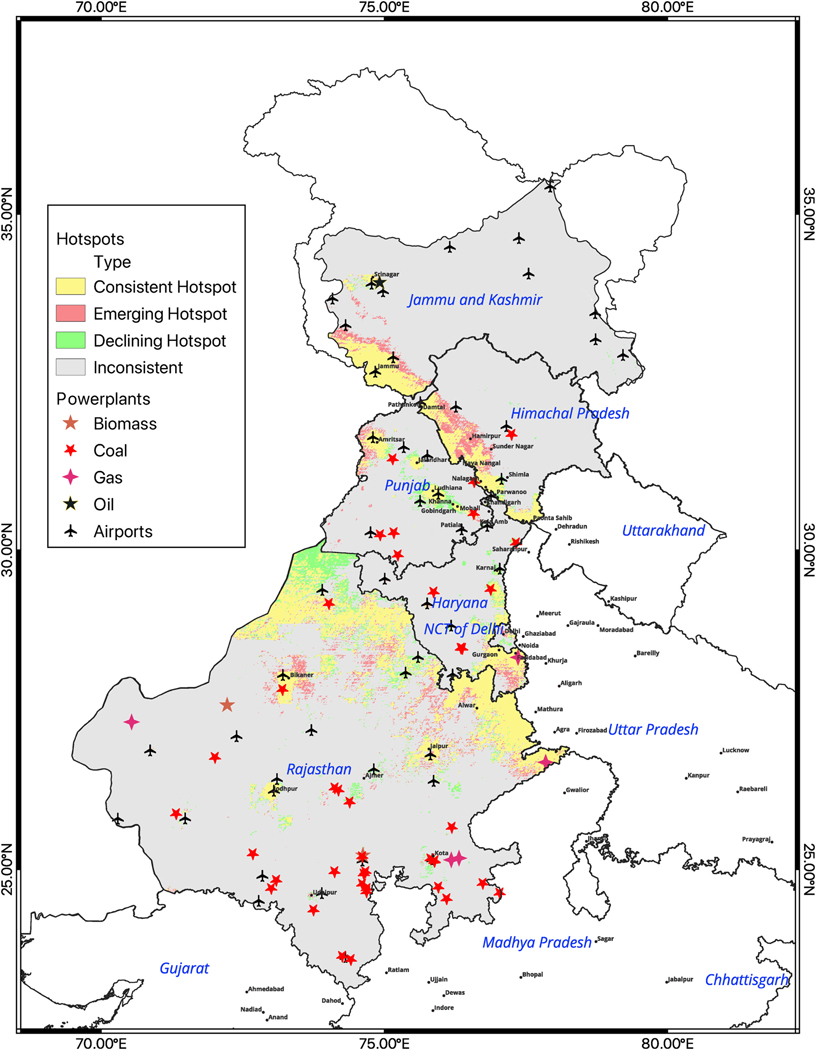

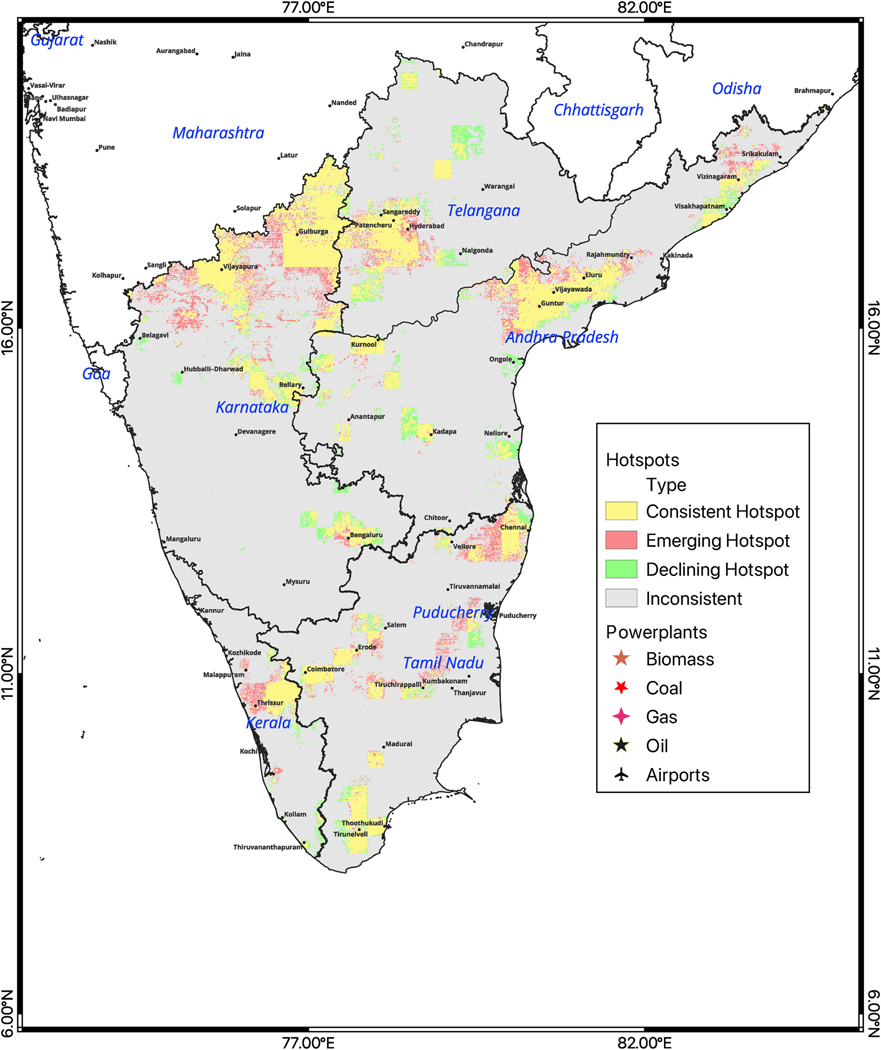

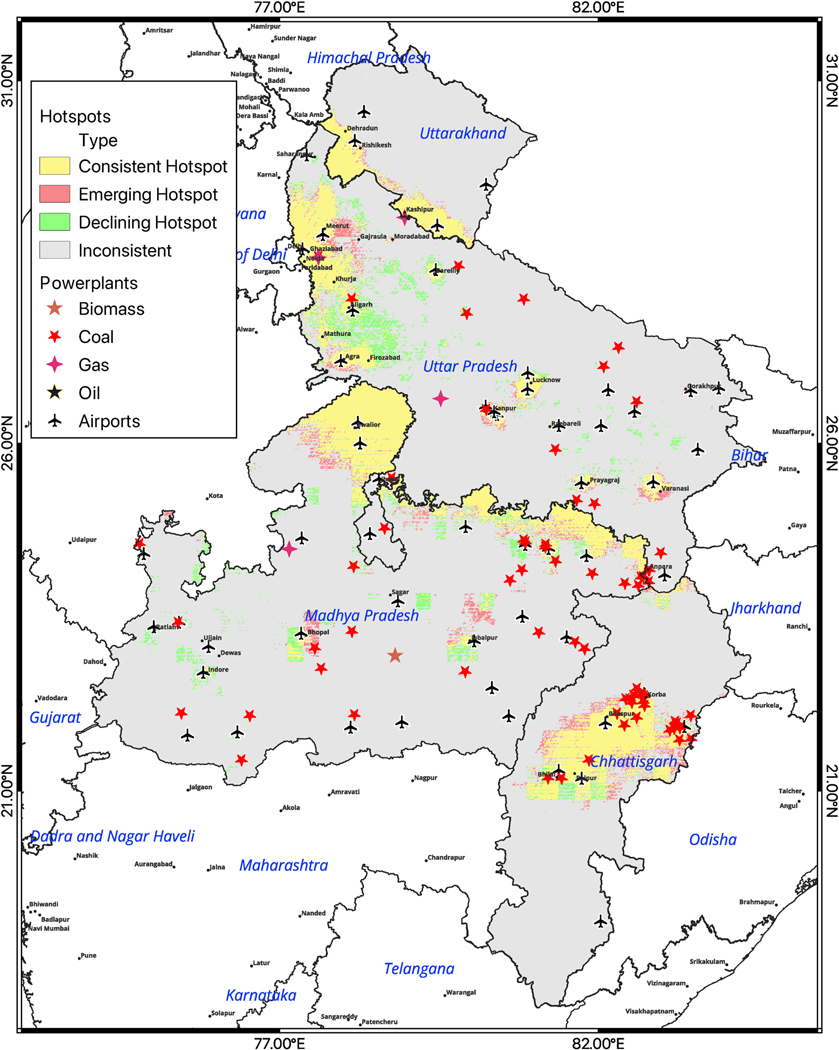
a:Hotspot trends, point sources of air pollution such as powerplants and airports in the states/UT of Rajasthan, Haryana, Punjab, Delhi, Himachal Pradesh & Jammu and Kashmir: North Zone b: Hotspot trends, point sources of air pollution such as powerplants and airports in the states/UT of Tamil Nadu, Telangana, Andhra Pradesh, Puducherry, Kerala & Karnataka: South Zone c: Hotspot trends, point sources of air pollution such as powerplants and airports, in the states of Uttarakhand, Uttar Pradesh, Madhya Pradesh & Chhattisgarh: Central Zone.

In the Northern Zone with states like Rajasthan, Haryana, Punjab, Himachal Pradesh and union territories like Chandigarh, Delhi & Jammu and Kashmir, the hotspot analyses identified a large area along the southern border of Jammu and Kashmir and Himachal Pradesh (amounting to 11% of this state’s/UT’s area) with a central core of consistent hotspots as well as a perimeter of emerging hotspots, pointing to an area of growing concern. Potential contributors to air pollution these areas could be lower elevation/valleys, industrial clusters, road networks (Road network) and heavy traffic, including related resuspended road dust, dispersion from neighboring states and anthropogenic activities air pollution settling in these regions. In all, Himachal Pradesh, Jammu and Kashmir, and Haryana had more than 20% of the population of the state/UT living in areas identified as consistent hotspots [Fig. 5a & Supplementary Table S1]. More than 25 non-major/metro cities (Location of major Indian cities, 2019) in the northern zone were identified as consistent/emerging hotspots. [Supplementary Table S2]. As an exception, Rajasthan had a large number of coal powered plants which were not in emerging or consistent hotspots, but we lack data whether on functional status of power plants during the study period. In Punjab, consistent hotspots were located along the major highways, including non-major/metro cities.

The Southern Zone consists of states like Tamil Nadu, Telangana, Andhra Pradesh, Kerala & Karnataka and union territory of Puducherry. The absolute PM_2.5_ levels were lower in the southern zone than in northern and central zones (Supplemental Table 1). We observed consistent and emerging hotspots around major urban areas in Hyderabad and Chennai as well as outside of major cities. Telangana had the highest concentrations of PM_2.5_ across all types of hotspots with 43.9 million people living in the state. Karnataka had the highest number of grid cells identified as consistent hotspots and were more densely populated than other states in the southern zone (14.4 million; 18.9% of the state’s population). Emerging hotspots were also highest in Karnataka and densely populated [5.3 million]. The northern border of Karnataka around Vijaypura and Gulbarga demonstrated a large area of consistent and emerging hotspots reflecting the influence of the neighboring state Maharashtra. Many gas- and coal-powered plants are located in consistent hotspots in Rajahmundry, Vijayawada, and Kakinada of Andhra Pradesh (Powerplants, 2024). The spatial distribution of declining hotspots did not demonstrate a clear pattern [Figure b & Supplementary Table S1]. It is worth noting that more than 22 consistent/emerging hotspot clusters were found in the non-major cities in the southern zone [Supplementary Table S2]. The southern zone has more than 200 powerplants including gas, coal, oil, nuclear and biomass (Powerplants, 2024). We observed a pattern of consistent and emerging hotspots around the powerplants in Northern Karnataka and Southern Tamil Nadu. Major highways and toll plazas are also often found close to consistent or emerging hotspots, for example, between Coimbatore and Salem in Tamil Nadu (Toll plazas; Road network). Emerging hotspots were found in the south-west stretch of Puducherry, a highway between Chennai and Tiruchirappalli with some non-major cities alongside some of the gas- and coal-powered plants (Powerplants, 2024). Kerala, that has cleaner air than other states, also had some consistent and emerging hotspots in cities like Palakkad, Thrissur, Malappuram and Thiruvananthapuram.

The Central Zone comprised of states like Uttarakhand, Uttar Pradesh, Madhya Pradesh & Chhattisgarh. Uttar Pradesh is the most populous state of India, squarely located in the Indo-Gangetic plain and had the highest concentrations of PM_2.5_ across all types of hotspots with - the lowest number of consistent hotspots yet the most densely populated emerging hotspots (5.4 million). [Fig. 5c & Supplementary Table S1]. Areas in Madhya Pradesh and Uttarakhand bordering on Uttar Pradesh had large clusters of consistent hotspots surrounded by emerging hotspots indicating an expanding area of high PM_2.5_ levels. In Madhya Pradesh, the areas around Bhopal and Jabalpur were identified as emerging hotspots without significant adjacent consistent hotspots, indicating new areas of increased exposure. Interestingly, hotspot analysis in Chhattisgarh, a mining state, demonstrated a large central area around Raipur and Bhilali with consistent and emerging hotspots bordering to 24 powerplants, mostly coal powered (Powerplants, 2024). The Central Zone had at least 25 non-major cities that were identified as consistent/emerging hotspots (Location of major Indian cities, 2019). [Supplementary Table S2]

We provide the hotspot trend maps for other zones such as West [Maharashtra, Gujarat, Daman & Diu, Dadra & Nagar Haveli & Goa], East [Bihar, Jharkhand, West Bengal & Odisha] and North-East [ Assam, Tripura, Meghalaya, Nagaland, Manipur, Sikkim, Arunachal Pradesh & Mizoram] in the supplement.

### 3.4. Hotspot trends across three mega cities: Delhi, Mumbai, and Kolkata metropolitan region

**Fig. 6.**
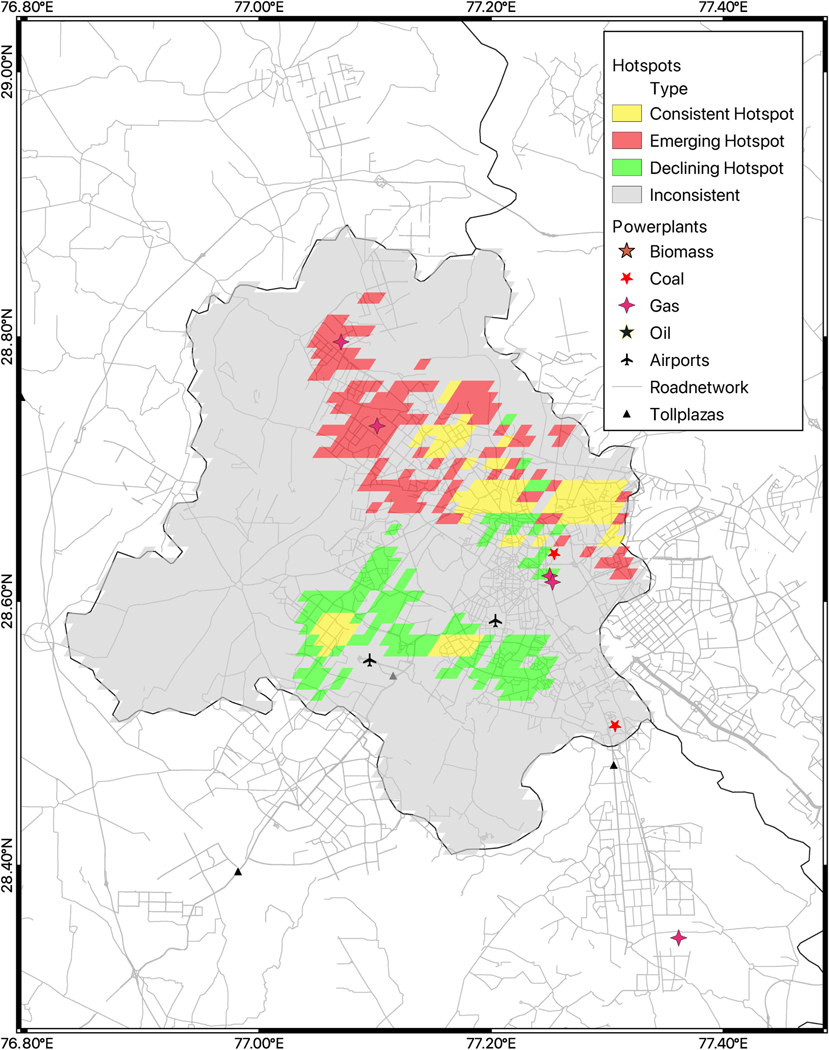

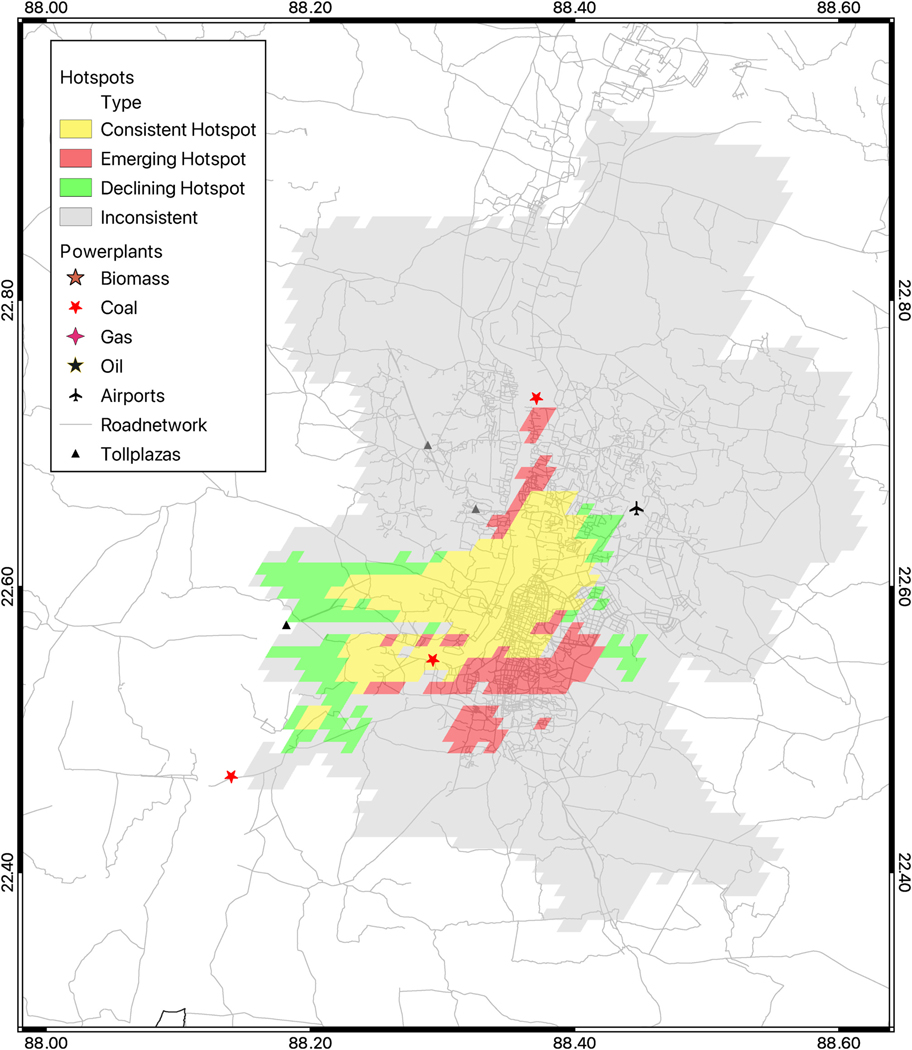

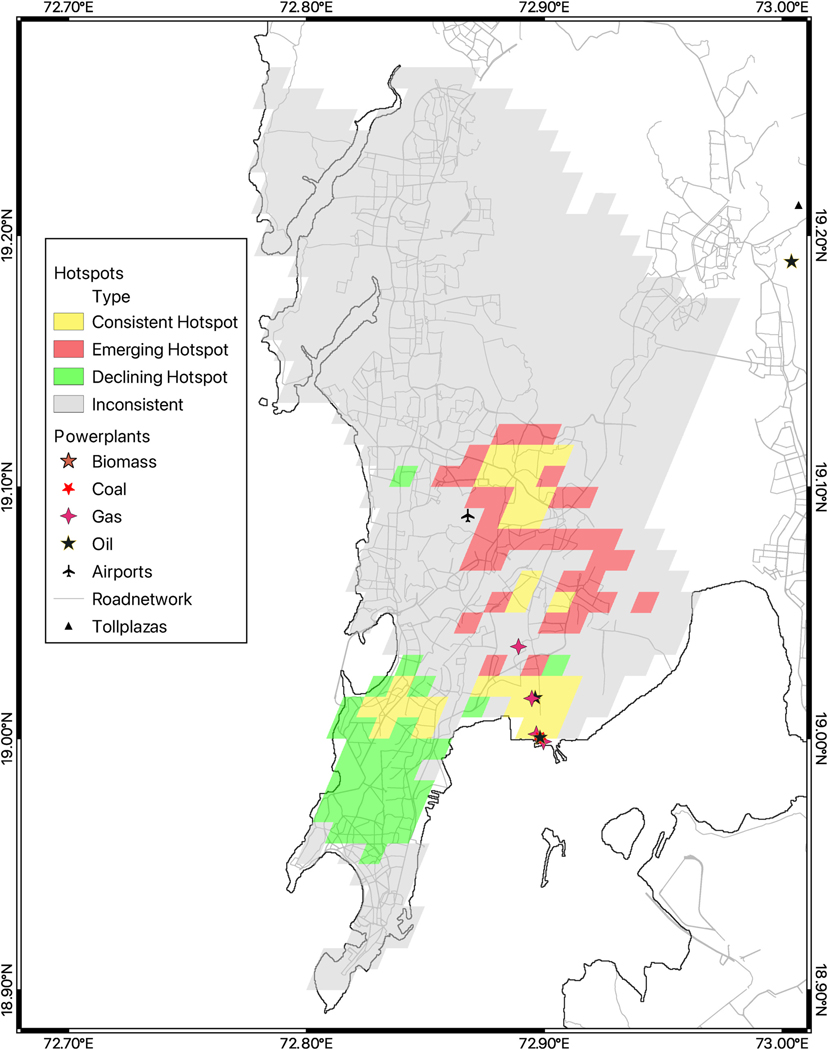
a, b & c: Hotspots trend maps, point sources of air pollution such as powerplants, airports, toll plazas and road network of Delhi (UT), Kolkata Metropolitan Region and Mumbai [2008–2019].

Among the three largest cities studied, we observed the highest PM_2.5_ concentrations in Delhi compared to all cities studied with 107.1 μg/m^3^ [13.6] for the 12-year average [SD]. All categories of hotspots in Delhi demonstrated concentrations *>*100 μg/m^3^ for the 12-year average. Delhi also had large proportion of grids identified as emerging hotspots among the three cities. Compared to the other cities Kolkata had more grids identified as consistent hotspots and included 20.4% of the city’s population (Fig. 6 a, b & Table 1). Some interesting spatial patterns of hotspots were noted. In Delhi, most of the consistent hotspots are centered around the densely populated old city center. A large area in the southern part of the city showed a promising clustering of declining hotspot grids, whereas emerging hotspots were observed in the central and northern part of the city. In Mumbai a similar south-north shift was observed with the southern part demonstrating a cluster of declining hotspot grids whereas emerging hotspots expanded around a cluster of consistent hotspots near the airport (Airports: David Megginson, 2008). In Kolkata, consistent hotspots were observed in the central area, with declining hotspots east and west of this area and emerging hotspots expanding north and south of the central area. Overlaying spatial locations of airports, toll plazas and powerplants in each city, we noted that 2 powerplants operating with gas as main fuel were located in the areas identified as emerging hotspots in Delhi (Toll plazas; Powerplants, 2024; Airports: David Megginson, 2008). Kolkata had two coal-fired power plants, each situated in consistent and emerging hotspots (Powerplants, 2024). Mumbai had 8 power plants (equal mix of oil and gas-fueled), and 7 of them were located in consistent hotspot areas (Powerplants, 2024). Mumbai airport was closer to consistent and emerging hotspots (Airports: David Megginson, 2008).

In summary, Delhi had extremely high PM_2.5_ levels, Kolkata had a substantial percentage of their population living in consistent hotspots while Mumbai had consistent hotspots with the lowest PM_2.5_ concentrations of the 3 megacities.

### 3.5. National clean air program (NCAP) cities and hotspots

The Ministry of Environment, Forest and Climate Change, Government of India launched the National Clean Air Programme (NCAP) (Ganguly et al., 2020) in 2019 as a long-term, time-bound, national-level strategy to tackle the air pollution problem across the country in a comprehensive manner. The NCAP targeted to achieve 20%–30% reduction in concentrations of PM_10_ and PM_2.5_ by the year 2024, keeping 2017 as the base year for comparison of concentration in 131 non-attainment cities (cities that consistently do not meet the National Ambient Air Quality Standards (NAAQS)). In our analysis that covers the period before the program launch, among the 131 NCAP cities, 85 were identified as consistent hotspots, 11 as declining, 17 as emerging and 18 as not hotspots. In the Supplementary Table S2, we provide details of other cities that are not listed in the NCAP but identified as consistent or emerging hotspots in our analyses. Of interest, we found in our analyses 119 cities and 51 cities as consistent and emerging hotspots, respectively, that could be considered for inclusion in the NCAP (Supplementary Table S2). This highlights the need to focus on shifting the interventions and air quality management to cover these non-major/metro cities too which may add to the burden of non-attainment cities in the future.

## 4. Discussion

Air pollution levels across India vastly exceed WHO air quality guidelines and to a large extent, the Indian national ambient air quality standards that are set 8 times higher for annual PM_2.5_ levels (*<*40 μg/m^3^). The highest levels are seen in the Indo-Gangetic plain that runs south of the Himalayas, which corresponds to the most densely populated subcontinent.

As a complement to the usual methods of estimating absolute levels of PM_2.5_ across India, our hotspot analysis identified areas within states/UTs and cities where levels were consistently higher compared to the rest of the state/UT or city and areas where levels showed a pattern of emerging or declining pollution zones. The significance of a consistent hotspot is that it represents an area where air pollution is high relative to other areas and stays high over time. These are areas that should be prioritized and targeted for interventions. Emerging hotspots are also areas to be targeted because they present a non-desirable trend. There could also be places where air pollution is improving, represented by the declining hotspots, which can serve as good case studies.

Our results also demonstrated the presence of consistent and emerging hotspots outside of major urban areas and in cities not currently included in the National Clean Air Programme of India. While levels of PM_2.5_ in India must be reduced across the board to reduce harmful health effects, this analysis provides important opportunities for policy makers for targeted interventions. To further characterize the hotspots, we also identified coal power plants and toll plazas as associated with these local hotspots. Coal burning powerplants in India often do not have bag houses (the most effective method to remove PM_2.5_ emissions) and generally do not have scrubbers, which remove the gases that produce particles in the atmosphere (Cropper et al., 2021). Toll plazas result in vehicle idling, increasing emissions, although a recent shift to Radio-frequency identification (RFID) based tolls has reduced waiting times. Hence, these are quite plausible sources of some hotspots, and solutions exist to ameliorate both sources.

Globally, similar studies have been conducted to identify air pollution hotspots. Abecasis et al., 2022 confirmed the influence of steel industries on air quality and the local population’s concerns. A study identified NO_2_ hotspots within the city using Getis-Ord $G_{i}^{*}$ statistic and exposing ~500, 000 people to NO_2_ concentrations that are 16%–32% higher than the city-wide average (Montgomery et al., 2023). A Chinese study examined air pollution [PM_2.5_] hotspots; the findings pinpointed some areas in the country as hotspots, similar to the present study (Ali et al., 2023).

In India, Dey et al. (2012) examined the presence of hotspots using the space–time variability of bias-corrected air pollution data from 2000 to 2010 on a regional level. The authors identified 5 hotspots at very coarse spatial resolution (17 km^2^) covering 11 Indian states and parts of Bangladesh housing 23% of the population of the country, where they observed an increase of *>*15 μg/m^3^ over the decade. Although helpful in raising awareness, the lack of more precise spatial reference at the city-level or state-level makes prioritizing actions to control air pollution difficult (Dey et al., 2012). In contrast, our study has identified areas of consistent, emerging, and declining hotspots using local indicators of spatial association (LISA), using Getis-Ord ${\text{Gi}}^{*}$ statistic for each state/UT and major cities from 2008 to 2019, which can help prioritize policy implementations for more targeted action.

A study conducted by Ruidas et al. (Ruidas and Pal, 2022) in Maharashtra using Getis-Ord $G_{i}^{*}$ statistic identified hotspots of air pollution and highlighted changes in conjunction with the covid-19 lockdown and reduced anthropogenic activities. An analysis conducted by Goyal et al. explored temporality of hotspots during 2018–2021 Delhi and indicated that there were a higher number of hotspots in April compared to May and June, while almost the entire city was a hotspot from October to February, in part due to the widespread crop-burning activities around the areas surrounding Delhi (Goyal et al., 2021). Zheng et al. found that a 10 μg/m^3^ difference in annual average PM_2.5_ was observed between the local hotspots and cool spots, indicating higher spatial variability and subsequent health inequalities due to long-term ambient PM_2.5_ exposures within a city like Delhi (Zheng et al., 2021). Our analysis extended the most common approach using Getis-Ord $G_{i}^{*}$ statistic to identify hotspots across the country, specific to each state/UT and three largest cities at annual level. We also analyzed the temporal trend for a 12-year period for all the studied spatial units.

Overall, our study extends the understanding of the air pollution problem in India by providing important insights into the location of hyperlocal high-pollution areas designated as consistent, emerging or declining between two selected time periods. The analysis for the first time in India demonstrated the spatio-temporal variation of pollution hotspots across all states/UTs and cities.

There are several motivations to identify air pollution hotspots. Firstly, to guide and ensure that the placement of the limited air pollution monitors across the cities and states/UTs captures relevant variations in exposure. Secondly, to guide implementation of interventions to reduce air pollution among these hotspots to protect human health, especially amongst vulnerable population groups. Thirdly, to track the progress of the interventions and to optimize the allocation of resources to prioritized interventions in identified hotspots. Hotspots can also be monitored for temporal trends in air pollution, and further guide research into the health impacts of air pollution in these areas.

## 5. Strengths and limitations

This analysis has several notable strengths. Firstly, we can pinpoint problematic areas/regions within each state/UT and city to aid in targeted policies to reduce air pollution. Secondly, these analyses can be replicated at various temporal scales (daily/monthly) and for any pollutant of interest. Thirdly, through this analytical tool, we can assess the effectiveness of policy intervention. Lastly, this analysis can identify rapidly changing areas that can be better monitored for air pollution. While mentioning the strengths of the analysis, we must also acknowledge that we are not able to pinpoint the real-time sources of air pollution, which future research studies can address. We performed these analyses at state/UT-level and city-level providing hotspots relative to the levels within each analytical spatial unit (state/UT or city). This induces some border effects, especially in state/UT-level analyses where hotspots were identified at the borders in states/UTs with lower mean levels of PM_2.5_ compared to neighboring states with higher mean levels of PM_2.5_ (where the border area might not be identified relative to its state’s level). Also, to capture temporal changes in hotspots, we arbitrarily compared the first half of our study period with the second half to simplify comparisons within each state/UT. This may have overlooked important local interventions or developments that could be the subject of a more targeted study using this approach.

## 6. Conclusion

To conclude, as a complement to assessing absolute levels of PM_2.5,_ we were able to identify hotspots of air pollution across India reflecting spatial clustering of high PM_2.5_ levels relative to the spatial unit of analysis using, a methodologically rigorous statistical approach that can help inform targeted action in air quality management and aid appropriate resource allocation. The insights from this work can also serve as a baseline for assessing the effectiveness of interventions and future programs and policies.

## Supplementary Material

Supplement file

## Figures and Tables

**Table 1:** Distribution of consistent, emerging, and declining hotspots across major cities in India: PM_2.5_ concentrations [12-year annual average], area coverage (%), and population in millions (%) [arranged in the descending order of PM_2.5_ concentrations in the consistent hotspots].

Cities Total population (m) Total area (grids)	Overall PM_2.5_ concentrations 12-y average [SD]	Consistent hotspot			Emerging hotspot			Declining hotspot			Inconsistent		
		PM_2.5_ [SD]	Area in grids (%)	Population in millions (%)	PM_2.5_ [SD]	Area in grids (%)	Population in millions (%)	PM_2.5_ [SD]	Area in grids (%)	Population in millions (%)	PM_2.5_ [SD]	Area in grids (%)	Population in millions (%)

Delhi [20.1m 1748g]	107.1 [13.6]	131.7 [14.1]	86 (4.9)	1.9 (9.4)	121.6 [9.1]	138 (7.9)	1.9 (9.4)	120.3 [9.6]	123 (7.1)	1.3 (6.6)	102.9 [14.4]	1401 (80.2)	14.9 (74.6)
Kolkata [20.1m 2250g]	50.2 [3.7]	62.5 [4.9]	188 (8.4)	4.1 (20.4)	58.3 [4.0]	84 (3.7)	1.7 (8.6)	55.2 [2.4]	98 (4.4)	1.7 (8.2)	48.4 [3.7]	1880 (83.6)	12.6 (62.8)
Mumbai [15.3m 531g]	41.8 [3.3]	47.1 [2.2]	31 (5.8)	1.1 (6.9)	45.8 [2.5]	34 (6.4)	0.9 (6.1)	44.8 [3.2]	34 (6.4)	1.4 (9.1)	40.9 [3.4]	432 (81.4)	11.9 (77.9)

## Data Availability

Data will be made available on request.

## Data sharing

All the data in this study are routinely collected and contain no information about specific people. Our data are available upon request to the corresponding author, subject to the agreement of the CHAIR-India Publications and Executive Committee.
