# Supplementary material for: Nationwide analysis of air pollution hotspots across India: A spatiotemporal PM2.5 trend analysis (2008–2019)
Source: Environ Res. Author manuscript; Available in PMC 2025 Feb 3. (PMC11790316; doi:10.1016/j.envres.2024.120276)
Supplement: Supplement file [file NIHMS2045243-supplement-Supplement_file.docx]

**Title.**

**Nationwide analysis of air pollution hotspots across India: a spatiotemporal PM2.5 trend analysis (2008-2019)**

**Authors:**

**Suganthi Jaganathan^1,2,3^, Ajit Rajiva^2,3,4^, Heresh Amini^5^, Jeroen de Bont^1^ ,Shweta Dixit^2,3^,Anubrati Dutta^3^, Itai Kloog^4,5^, Kevin J. Lane^6^, Jyothi S Menon^2,3^, Amruta Nori-Sarma^6^, Dorairaj Prabhakaran^3,7^, Joel Schwartz^8^, Praggya Sharma^3^, Massimo Stafoggia^1,9^,Gagandeep Kaur Walia^2,3^, Gregory A. Wellenius^6^, Poornima Prabhakaran^2,3^, Petter Ljungman^1,10*^, Siddhartha Mandal^2,3*^**

**Affiliations:**

1- Institute of Environmental Medicine, Karolinska Institutet, Stockholm, Sweden

2-Centre for Health Analytics Research and Trends, Ashoka University, Sonipat, Haryana, India

3-Centre for Chronic Disease Control, Delhi-NCR, India

4-Department of Geography and Environment, Faculty of Humanities and Social Sciences, Ben-Gurion University of the Negev, Beer-Sheva, Israel

5-Department of Environmental Medicine and Public Health, Icahn School of Medicine at Mount Sinai, New York, NY, USA

6-Department of Environmental Health, School of Public Health, Boston University, Boston, MA, USA

7-Public Health Foundation of India

8-Department of Environmental Health, Harvard T.H. Chan School of Public Health, Boston, MA, USA

9-Department of Epidemiology, Lazio Region Health Service / ASL Roma 1, Rome, Italy

10-Department of Cardiology, Danderyd University Hospital, 182 57, Danderyd, Sweden

*shared last authors

| **Supplementary table S1. Distribution of consistent, emerging, and declining hotspots across the states/UTs in India: PM2.5 concentrations [12-year annual average], area coverage (%), and population in millions (%) [arranged in the descending order of PM2.5 concentrations in the consistent hotspots]** | | | | | | | | | | | | |
| --- | --- | --- | --- | --- | --- | --- | --- | --- | --- | --- | --- | --- |
| **States/UTs**  **Total area(grids)**  **Total population(m)** | **Consistent hotspot** | | | **Emerging hotspot** | | | **Declining hotspot** | | | **Inconsistent** | | |
|  | **PM2.5 [SD]** | **Area in grids (%)** | **Population in millions (%)** | **PM2.5 [SD]** | **Area in grids (%)** | **Population in millions (%)** | **PM2.5 [SD]** | **Area in grids (%)** | **Population in millions (%)** | **PM2.5 [SD]** | **Area in grids (%)** | **Population in millions (%)** |
| **Northern Zone** | | | | | | | | | | | | |
| Haryana  51335g  31.58m | 82.6  [10.6] | 5026 (9.8) | 7.3  (23.2) | 72.9  [3.3] | 987 (1.9) | 0.8  (2.4) | 71.6  [4.1] | 1037 (2.0) | 0.9  (2.7) | 58.2  [6.7] | 44285 (86.3) | 22.64 (71.7) |
| Punjab  58853g  34.51m | 68.4  [8.2] | 3627 (6.2) | 4.02  (11.7) | 60.7  [2.7] | 1119 (1.9) | 0.7  (1.9) | 59.6  [3.0] | 1710 (2.9) | 1.5  (4.3) | 52.8  [4.4] | 52397 (89.1) | 28.3 (82.1) |
| Rajasthan  399527g  86.62m | 59.2  [4.5] | 41813 (10.5) | 15.62 (18.1) | 54.9  [2.4] | 8142 (2.1) | 2.6  (2.9) | 54.9  [2.5] | 10752 (2.7) | 2.9  (3.3) | 48.2  [3.9] | 338820 (84.8) | 65.5 (75.7) |
| Chandigarh  139g  0.91m | 49.3  [2.2] | 10  (7.2) | 0.07  (7.4) | 48.4  [2.6] | 6  (4.3) | 0.04  (4.5) | 48.7  [3.9] | 6  (4.3) | 0.04  (4.5) | 44.2  [2.9] | 117 (84.2) | 0.8 (83.6) |
| Jammu & Kashmir  124266g  16.22m | 43.9  [6.0] | 7268 (5.9) | 3.6  (22.3) | 33.9  [2.5] | 2237 (1.8) | 0.5  (3.0) | 32.9  [2.1] | 179  (0.1) | 0.09  (0.5) | 27.3  [2.6] | 114582 (92.2) | 12.1 (74.2) |
| Himachal Pradesh  64929g  8.69m | 41.5  [4.4] | 7136 (10.99) | 2.35  (26.99) | 36.5  [2.2] | 3454 (5.3) | 1.3  (15.1) | 34.9  [3.1] | 319  (0.5) | 0.1  (1.1) | 27.0  [3.4] | 54020 (83.2) | 4.9 (56.9) |
| **Western** **Zone** | | | | | | | | | | | | |
| Maharashtra  359311g  141.92m | 44.9  [4.4] | 29544 (8.2) | 20.2  (14.2) | 41.1  [2.3] | 10918 (3.1) | 16.9 (11.9) | 41.0  [2.7] | 11826 (3.3) | 4.9  (3.4) | 35.3  [4.1] | 307023 (85.5) | 99.9 (70.4) |
| Gujarat  216724g  77.23m | 44.4  [2.5] | 30775 (14.2) | 9.7  (12.6) | 42.2  [1.7] | 3885 (1.8) | 1.1  (1.4) | 41.1  [2.1] | 4816 (2.2) | 2.64  (3.4) | 36.2  [3.2] | 177248 (81.8) | 63.8 (82.6) |
| Daman & Diu  113g  0.23m | 37.5  [1.2] | 6  (5.3) | 0.01  (6.1) | 36.1  [0.8] | 2  (1.8) | <0.01  (2.1) | 36.5  [1.2] | 5  (4.4) | 0.01  (5.1) | 32.4  [3.8] | 100 (88.5) | 0.2  (86.8) |
| Dadra & Nagar Haveli  575g  0.49m | 36.7  [0.8] | 60 (10.4) | 0.02  (4.4) | - | - | - | 35.6  [0.9] | 15  (2.6) | 0.01  (2.8) | 33.9  [1.5] | 500 (86.9) | 0.46 (92.8) |
| Goa  4309g  1.88m | 25.3  [1.9] | 104 (2.4) | 0.02  (1.2) | 22.5  [1.1] | 419 (9.5) | 0.1  (7.4) | 21.8  [1.6] | 465 (10.6) | 0.2  (10.9) | 19.9  [1.5] | 3402 (77.5) | 1.5 (80.5) |
| **Southern Zone** | | | | | | | | | | | | |
| Telangana  133972g  43.93m | 41.3  [4.0] | 11318 (8.5) | 6.6  (15.1) | 38.1  [2.0] | 2770 (2.1) | 4.6  (10.5) | 37.4  [1.7] | 3828 (2.9) | 1.4  (3.2) | 32.5  [3.2] | 116056 (86.6) | 31.3 (71.2) |
| Tamil Nadu  152887g  91.08m | 35.8  [3.5] | 13991 (9.2) | 13.7 (15.1) | 32.6  [1.5] | 5657 (3.7) | 4.4  (4.9) | 32.4  [1.9] | 2883 (1.9) | 2.6  (2.9) | 27.1  [3.3] | 130356 (85.3) | 70.3 (77.2) |
| Andhra Pradesh  187564g  60.86m | 34.7  [1.9] | 20458 (10.9) | 11.1 (18.3) | 32.5  [1.3] | 5472 (2.9) | 2.4  (4) | 32.4  [1.6] | 5249  (2.8) | 2.5  (4.1) | 28.4  [2.5] | 156385 (83.4) | 44.8  (73.6) |
| Karnataka  223742g  75.87m | 34.5  [2.6] | 28455 (12.7) | 14.4  (18.9) | 31.4  [1.8] | 9587 (4.3) | 5.3  (7.0) | 31.5  [1.8] | 4326 (1.9) | 1.82  (2.4) | 25.2  [4.2] | 181374 (81.1) | 54.3 (71.6) |
| Kerala  44434g  40.06m | 26.4  [2.5] | 3667 (8.3) | 3.0  (7.5) | 23.4  [1.0] | 1829 (4.1) | 2.0  (5.1) | 23.1  [1.2] | 1049 (2.4) | 0.78  (1.9) | 19.5  [2.5] | 37889 (85.3) | 34.2 (85.5) |
| Puducherry  668g  1.4m | 26.2  [1.7] | 52  (7.8) | 0.05  (3.7) | 24.8  [0.9] | 22  (3.3) | 0.05  (3.7) | 25.6  [3.5] | 5  (0.8) | 0.01  (1.0) | 19.6  [2.5] | 589 (88.2) | 1.3 (91.6) |
| **Central Zone** | | | | | | | | | | | | |
| Uttar Pradesh  281255g  252.34m | 83.0  [9.7] | 20858 (7.4) | 38.9 (15.5) | 74.3  [4.3] | 3801 (1.4) | 5.4  (2.1) | 72.9  [2.9] | 11087 (3.9) | 10.8  (4.3) | 62.6  [7.3] | 245509 (87.3) | 197.2 (78.1) |
| Uttarakhand  63182g  13.13m | 56.1  [5.6] | 9140 (14.5) | 6.4  (48.9) | 45.1  [3.5] | 627 (0.9) | 0.3  (1.9) | 45.4  [4.1] | 109  (0.2) | 0.03  (0.2) | 29.3  [5.5] | 53306 (84.4) | 6.4  (48.9) |
| Madhya Pradesh  359513g  91.56m | 55.5  [5.1] | 33432 (9.3) | 13.0 (14.2) | 49.1  [2.5] | 5531 (1.5) | 2.2  (2.3) | 47.9  [3.1] | 8856 (2.5) | 3.8  (4.1) | 41.3  [4.2] | 311694 (86.7) | 72.6 (79.3) |
| Chhattisgarh  158133g  32.05m | 50.5  [4.2] | 23100 (14.6) | 10.7 (33.3) | 46.1  [1.9] | 4435 (2.8) | 1.35  (4.2) | 45.6  [2.7] | 2384 (1.5) | 1.1  (3.3) | 34.2  [6.9] | 128214 (81.1) | 18.9 (59.2) |
| **Eastern Zone** | | | | | | | | | | | | |
| Bihar  110463g  135.46m | 65.8  [5.8] | 10698 (9.7) | 19.8 (14.7) | 61.4  [2.4] | 1821 (1.7) | 2.6  (1.9) | 60.9  [1.9] | 5217 (4.7) | 6.7  (4.9) | 52.4  [6.2] | 92727 (83.9) | 106.4 (78.5) |
| Jharkhand  93427g  42.44m | 60.7  [6.3] | 6824 (7.3) | 8.5  (20.1) | 54.9  [1.9] | 4627  (4.9) | 2.5  (5.9) | 54.0  [2.7] | 1665  (1.8) | 0.9  (2.3) | 45.9  [5.4] | 80311  (85.9) | 30.5  (71.7) |
| West Bengal  102102g  116.86m | 58.1  [5.9] | 9137 (8.9) | 15.8  (13.5) | 51.1  [3.4] | 1813 (1.8) | 10.4  (8.9) | 50.9  [2.6] | 2333 (2.3) | 1.9  (1.6) | 40.6  [6.4] | 88819 (86.9) | 88.76 (75.9) |
| Odisha  152887g  91.08m | 35.8  [3.5] | 13991 (9.2) | 13.7 (15.1) | 32.6  [1.5] | 5657 (3.7) | 4.4  (4.9) | 32.4  [1.9] | 2883 (1.9) | 2.6  (2.9) | 27.1  [3.3] | 130356 (85.3) | 70.3 (77.2) |
| **North-eastern Zone** | | | | | | | | | | | | |
| Assam  91808g  39.2m | 41.5  [3.1] | 11479 (12.5) | 7.7  (19.7) | 37.9  [2.6] | 3409 (3.7) | 2.1  (5.3) | 37.9  [2.5] | 2948 (3.2) | 1.7  (4.2) | 29.4  [5.8] | 73972 (80.6) | 27.75 (70.8) |
| Tripura  12712g  5.04m | 39.5  [3.8] | 815 (6.4) | 0.9  (17.1) | 31.3  [1.6] | 255 (2.0) | 0.2  (2.9) | 31.6  [2.2] | 33  (0.3) | 0.04  (0.7) | 25.0  [3.2] | 11609 (91.3) | 3.99 (79.2) |
| Meghalaya  26405g  4.43m | 37.6  [4.8] | 2095 (7.9) | 0.7  (15.5) | 30.6  [2.3] | 1294 (4.9) | 0.4  (9.0) | 29.3  [4.8] | 105  (0.4) | 0.02  (0.5) | 22.3  [3.2] | 22911 (86.8) | 3.32 (74.9) |
| Nagaland  19499g  2.29m | 30.4  [3.4] | 1765 (9.1) | 0.5  (19.8) | 26.2  [2.0] | 354 (1.8) | 0.04  (1.9) | 25.1  [1.5] | 116  (0.6) | 0.02  (0.9) | 20.6  [2.0] | 17264 (88.5) | 1.8 (77.5) |
| Manipur  26312g  3.73m | 27.4  [3.2] | 2280 (8.7) | 1.6  (42.2) | 24.5  [1.8] | 414 (1.6) | 0.2  (5.6) | 23.3  [1.7] | 343  (1.3) | 0.08  (2.0) | 19.2  [1.6] | 23275 (88.5) | 1.9 (50.2) |
| Sikkim  8506g  0.75m | 27.4  [1.7] | 579 (6.8) | 0.02  (2.7) | 25.6  [2.0] | 315  (3.7) | 0.08  (11.3) | 25.7  [1.5] | 799  (9.4) | 0.01  (0.7) | 22.4  [2.5] | 6813 (80.1) | 0.6 (85.3) |
| Arunachal Pradesh  95773g  1.82m | 24.4  [1.5] | 9010 (9.4) | 0.08  (4.4) | 22.5  [2.2] | 3427 (3.6) | 0.3  (16.1) | 22.7  [1.1] | 7763 (8.1) | 0.04  (2.1) | 19.2  [1.8] | 75573 (78.9) | 1.4 (77.4) |
| Mizoram  25077g  1.45m | 23.8  [1.9] | 1064 (4.2) | 0.13  (8.7) | 20.5  [1.1] | 243 (0.9) | 0.01  (0.9) | 19.5  [1.3] | 200  (0.8) | 0.02  (1.5) | 17.8  [0.9] | 23570 (93.9) | 1.3 (88.8) |

**Figure S1:** Hotspot trends, point sources of air pollution such as powerplants and airports, in the states/UTs like Maharashtra, Gujarat, Daman & Diu, Dadra & Nagar Haveli & Goa: West Zone


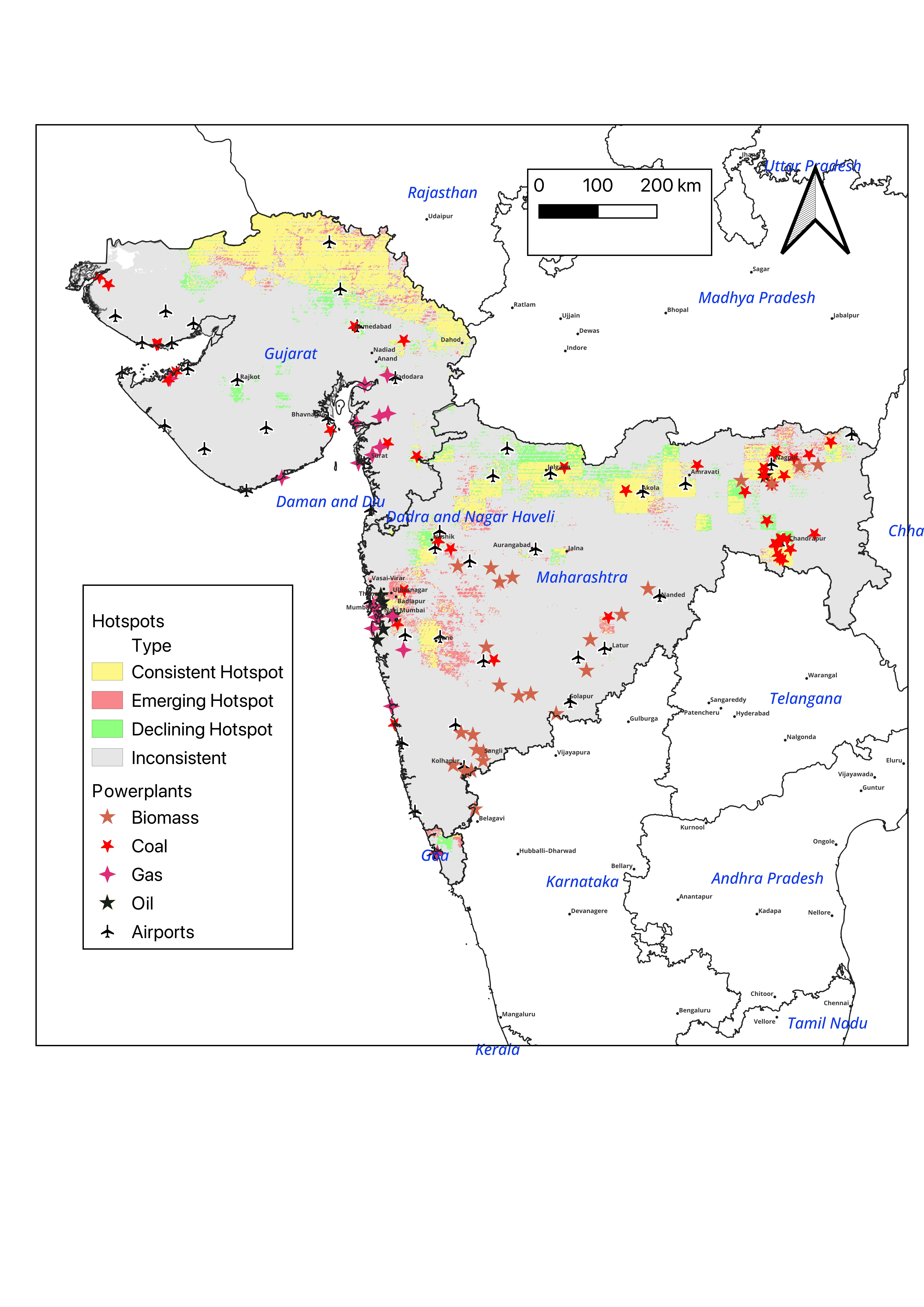


**Figure S2:** Hotspot trends, point sources of air pollution such as powerplants and airports, in the states like Bihar, Jharkhand, West Bengal & Odisha: East Zone


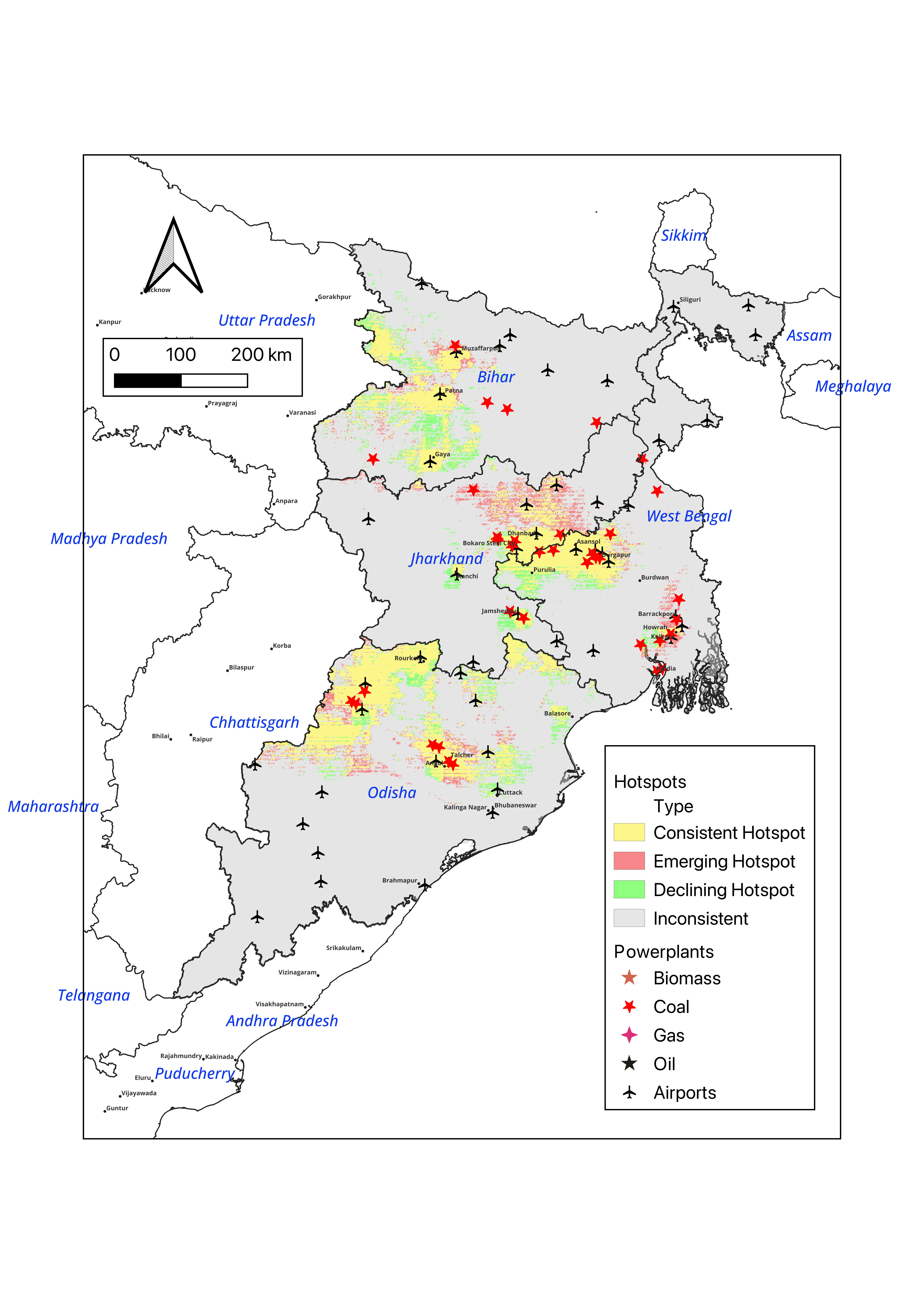


**Figure S3:** Hotspot trends, point sources of air pollution such as powerplants and airports, in the states like Assam, Tripura, Meghalaya, Nagaland, Manipur, Sikkim, Arunachal Pradesh & Mizoram: North-East Zone


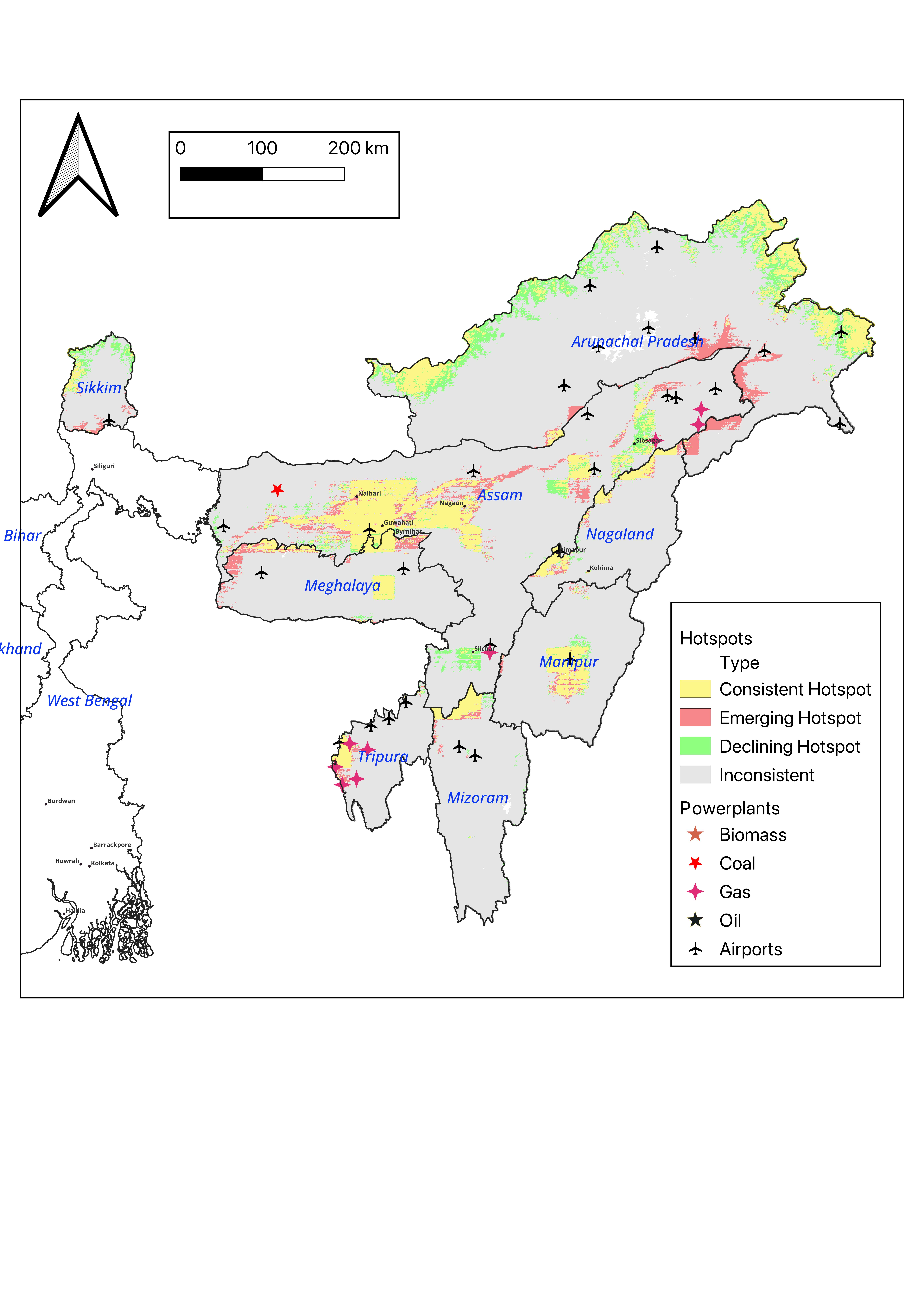


| **Supplementary table S2: National Clean Air Programme (NCAP) cities and other cities identified consistent and emerging hotspots in India but not included in the NCAP.** | | | | | | |
| --- | --- | --- | --- | --- | --- | --- |
| **#** | **State** | **NCAP cities** | **Count** | **Consistent hotspots** | **Count** | **Emerging hotspots** |
| 1 | Andhra Pradesh | Guntur, Kurnool, **Nellore**, Vijayawada, **Vishakhapatnam**, Anantapur, **Chitoor**, Eluru, Kadapa, **Ongole**, Rajahmundry, Srikakulam, Vizianagaram | 5 | Uravakonda, Aluru, Gudur, Banaganapalle, Chilkaluripet | 3 | Velduthri, Peapully, Parvathipuram |
| 2 | Assam | Guwahati, Nagaon, Nalbari, **Sibsagar, Silchar** | 5 | Goalpara, Dhubri, Deragaon, Tezpur, Kamrup | 2 | Goalghat, Dibrugarh |
| 3 | Bihar | Patna, Gaya, Muzaffarpur | 3 | Arrah, Chhapra, Siwan |  |  |
| 4 | Chandigarh | Chandigarh |  |  |  |  |
| 5 | Chattisgarh | Bhilai, Korba, Raipur | 3 | Bilaspur, Raigarh, Rajnandgaon |  |  |
| 6 | Delhi | Delhi |  |  |  |  |
| 7 | Gujarat | **Surat,** Ahmedabad, **Vadodara**, **Rajkot** | 6 | Goiteshwar, Palanpur, Deesa, Kachchh, Patan, Mahesana |  |  |
| 8 | Haryana | Faridabad | 12 | Kurukshetra, Karnal, Hisar, Panipat, Sonipat, Rohtak, Gurugram, Manesar, Rewari, Palwal, Sirsa, Ballabgarh |  |  |
| 9 | Himachal Pradesh | Baddi, Damtal, Kala Amb, Nalagarh, Paonta Sahib, **Parwanoo**, Sunder Nagar |  |  |  |  |
| 10 | Jammu & Kashmir | Jammu, Srinagar | 5 | Kulgam, Gulmarg, Kathua, Ramgarh, Samba | 2 | Udhampur, Rajouri |
| 11 | Jharkhand | Dhanbad, Jamshedpur, Ranchi | 3 | Giridih, Bokaro Steel City, Deoghar |  |  |
| 12 | Karnataka | Bangalore, Devanagere, Gulburga, **Hubli-Dharwad** | 7 | Tumakuru, Hosapet, Bellary, Raichur, Vijaypura, Bidar, Koppala | 4 | Mahalingapura, Lokapura, Mudhol, Kushtagi |
| 13 | Madhya Pradesh | Bhopal, **Dewas**, Indore, **Sagar, Ujjain**, Gwalior, Jabalpur | 5 | Sehore, Bhind, Morena, Rewa, Singaruli, | 2 | Damoh, Katni |
| 14 | Maharashtra | Akola, Amravati, **Aurangabad, Badlapur**, Chandrapur, Jalgaon, Jalna, Kolhapur, **Latur**, Mumbai, Nagpur, Nashik, Navi Mumbai, Pune, **Sangli, Solapur,** Ulhasnagar, Thane, Vasai-Virar | 5 | Pimpri-chinchwad, Malegaon, Dhule, Nandgaon, Gadchandur | 4 | Ambajogai, Kalyan, Dhawalpuri, Tumsar |
| 15 | Meghalaya | Byrnihat | 3 | Jirang, Shillong, East Khasi District | 1 | Southwest Garo Hills |
| 16 | Nagaland | Dimapur, Kohima | 3 | Changpang, Tuli, Namsang | 3 | Medziphema, Ngwalwa, Jaboka |
| 17 | Odisha | Angul, Balasore, **Bhubaneswar**, **Cuttack**, Rourkela, Talcher, **Kalinga Nagar** | 3 | Bargarh, Sundargarh, Sambalpur | 1 | Kendujhar |
| 18 | Punjab | **Dera Bassi,** Gobindgarh, Jalandhar, Khanna, Ludhiana, **Naya Nangal**, Pathankot/Dera Baba, **Patiala**, Amritsar | 6 | Batala, Tarn Taran Sahib, Kapurthala, Moga, Goindval Shaib,Patti | 8 | Rajpura, Sri Muktsar Sahib, Faridkot, Bathinda, Jalandhar, Fazilika, Abohar, Firozpur |
| 19 | Rajasthan | Alwar, Jaipur, Jodhpur, Kota, Udaipur | 8 | Bikaner, Jaitsar, Sri Ganganagar, Badhra, Churu, Bharatpur, Hanumangarh, Jhunjhunu |  |  |
| 20 | Tamilnadu | Thoothukudi, Trichy, Madurai, Chennai | 15 | Coimbatore, Erode, Sankagiri, Trippur, Vijayamangalam, Mettur, Neyveli, Karur, Triumangalam, Kovil patti, Kayathar, Triuneveli, Thotthukudi, Nanguneri, Vellore | 11 | Triuchengode, Omalur, Perambalur, Ulundurpettai, Villupuram, Ariyalur, Trichy, Sivakasi, Kanchipuram, Arakkonam, Chengalpattu |
| 21 | Telangana | Hyderabad, **Nalgonda**, Patancheruvu, Sangareddy | 6 | Siddipet, Kadthal, Mahabubnagar,Sultanbad, Choppadandi, Adilabad | 3 | Vikrabad, Tanduru, Choutupal |
| 22 | Uttar Pradesh | Agra, Allahabad, **Anpara**, Bareily, Firozabad, Gajraula, Ghaziabad, **Jhansi**, Kanpur, Khurja, Lucknow, Moradabad, Noida, Raebareli, Varanasi, Gorakhpur, Meerut | 6 | Aligarh, Mathura, Bulandshahr, Shamli, Saharanpur, Unnao | 2 | Sambhal, Mughal Sarai |
| 23 | Uttarakhand | Kashipur, Rishikesh, Dehradun | 3 | Roorkee, Haldwani, Rudrapur |  |  |
| 24 | West Bengal | Kolkata, Asansol, Barrackpore, Durgapur, **Haldia**, Howrah | 3 | Purunia, Berhampore, Bolpur |  |  |
| 25 | Kerala | NA | 4 | Palakkad, Chittur, Kollam, Thiruvananthapuram | 5 | Thrissur, Wadakancheri, Malappuram, Mannarkad, Kottayam |
|  |  |  | 119 |  | 51 |  |
| Note: **Green**-declining hotspots; **blue**- not a hotspot | | | | | | |
